# Putative Identification of 47 Compounds from *Jieyu Anshen* Granule and Proposal of Pharmacopeia Quality‐Assessment Strategy Using TCM‐Specific Library with UHPLC‐Q‐Exactive‐Orbitrap‐MS

**DOI:** 10.1002/open.202400046

**Published:** 2024-11-21

**Authors:** Xican Li, Jingyuan Zeng, Rongxin Cai, Chunhou Li, Xiaoshan Chen, Ban Chen, Xiaojun Zhao, Sunbal Khan

**Affiliations:** ^1^ School of Chinese Herbal Medicine Guangzhou University of Chinese Medicine Waihuan East Road No. 232, Guangzhou Higher Education Mega Center Guangzhou 510006 China; ^2^ Department of Pharmacy Guangdong Provincial People's Hospital Guangdong Academy of Medical Sciences) Southern Medical University Guangzhou 510006 China; ^3^ College of Pharmacy Chengdu University of Traditional Chinese Medicine Chengdu 611137 China; ^4^ Key Laboratory of Fermentation Engineering (Ministry of Education) Cooperative Innovation Center of Industrial Fermentation (Ministry of Education & Hubei Province) Hubei University of Technology Wuhan 430068 China; ^5^ Department of Chemistry University of Malakand Chakdara 18800, Dir Lower, Khyber Pakhtunkhwa Pakistan

**Keywords:** Adulterate, Chinese medicine, Glycyrrhizic acid, Saikosaponin A, Saikosaponin D, UPLC−Q- Orbitrap-MS/MS

## Abstract

*Jieyu Anshen* Granule is a traditional Chinese medicine prescription used for depression and primarily comprises five herbal medicines: Zhizi, Chaihu, Zhigancao, Danggui, and Yuanzhi. This study established a traditional Chinese herbal medicine‐specific library using emerging ultra‐high‐performance liquid chromatography‐quadrupole‐orbitrap‐tandem mass spectrometry analysis. Through library comparison, the study has fulfilled isomers distinction. As a result, 47 compounds were simultaneously and putatively identified from *Jieyu Anshen* Granule, including 12 unexpected compounds and 35 expected compounds. The unexpected compounds comprised cyclocommunol, 5‐hydroxyflavone, tangeretin, 3,5,6,7,8,3’,4’‐heptemethoxyflavone, calycosin‐7‐*O*‐β‐D‐glucoside, 7,4’‐dihydroxyflavone, naringenin‐7*‐O‐*β‐D‐glucoside, matrine, betaine, jervine, alantolactone, and hypericin. Among the 35 expected compounds, saikosaponin A, saikosaponin D, glycyrrhizic acid, geniposide, ligustilide, and polygalaxanthone III were further investigated using a quantum chemistry approach. Based on these, an effective quality assessment strategy is proposed for the Pharmacopeia, involving the simultaneous analysis of glycyrrhizic acid, geniposide, ligustilide, polygalaxanthone III, saikosaponins A and D through ultra‐high‐performance liquid chromatography‐quadrupole‐orbitrap‐tandem mass spectrometry analysis. This strategy enables the detection of adulteration in relation to Zhizi, Chaihu, Zhigancao, Danggui, and Yuanzhi in *Jieyu Anshen* Granule. The findings of unexpected compounds will deepen the understanding of chemistry in *Jieyu Anshen* Granule.

## Graphical abstract

Traditional Chinese medicine prescription *Jieyu Anshen* Granule is analyzed using UHPLC−Q‐Exactive Orbitrap‐MS/MS. Forty‐seven compounds are identified and 12 compounds are found in the Granule for the first time. Five compounds are proposed as Pharmacopeia quality‐markers.

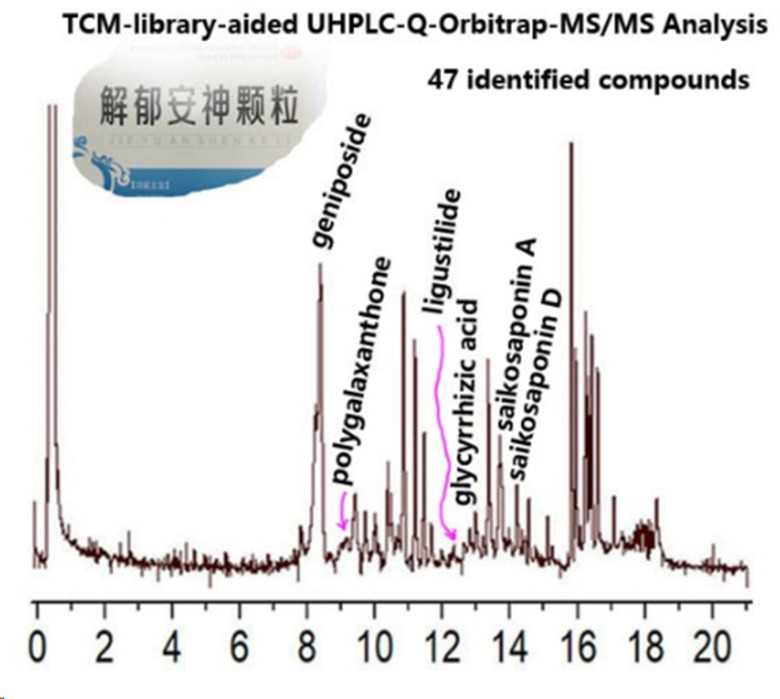

## Introduction

**Figure 1:**
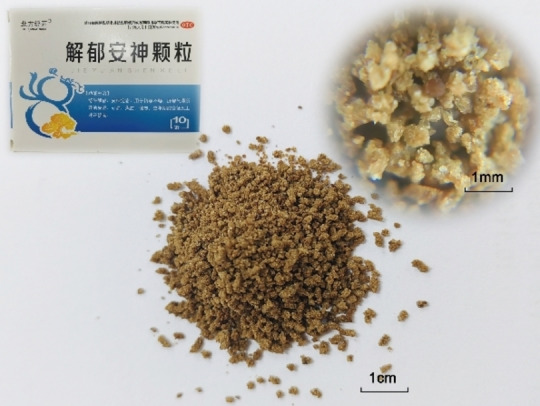
*Jieyu Anshen* Granule appearance (the insert upper right is an enlarged view).

Traditional Chinese medicine (TCM) has been used for centuries in China and other Asian countries, such as Korea and Japan, and is becoming popular worldwide for its ability to prevent and treat a variety of diseases. The Chinese term “Jieyu Anshen” refers to the process of relieving the psychological burden associated with depression and anxiety. In recent years, Jieyu Anshen Granule (Figure 1) is applied to treat various depression and anxiety, especially post‐stroke depression, hypnosis, and amnesia in TCM.^[1]^

Due to its proven efficacy and extensive use in TCM treatments, there are currently 40 pharmaceutical factories manufacturing *Jieyu Anshen* Granule, as reported by the National Medical Products Administration of China. Their manufacturing techniques are believed to adhere to the guidelines outlined in the Chinese Pharmacopeia (2020). As stated in the Chinese Pharmacopeia, this prescription consists of a mixture of 16 traditional Chinese herbal medicines (TCHMs), including Gardeniae fructus (Zhizi), Bupleuri radix (Chaihu), Glycyrrhizae radix et rhizoma praeparata cum melle (Zhigancao), Angelicae sinensis radix (Danggui), and Polygalae radix (Yuanzhi) (Table 1).^[2]^ In TCM, however, the mixing was based on the Monarch‐Minister‐Assistant‐Guide theory,^[3]^ while Chaihu and Zhigancao serving as the ‘Monarch’ (Jun Yao) and ‘Guide’ (Shi Yao) roles within the entire granule formulation, respectively. As the ‘Monarch’, Chaihu has the effect of soothing liver and relieving depression; while the ‘Guide’ Zhigancao can harmonize the actions of all medicines in a whole formula.

**Table 1:** The main information regarding *Jieyu Anshen* Granule and its formula.

Chinese name	Plant‐derived TCHMs	Mass /g	Pharmacopeia assessment	
			Q‐marker	Method
Zhizi	*Gardeniae Fructus*	80	Geniposide	HPLC
Chaihu	*Bupleurum chinense*	80	Saikosaponins A and D	HPLC
Zhigancao	*Glycyrrhizae radix* et *rhizoma praeparata* cum melle	60	Liquiritin and glycyrrhizic acid	HPLC
Danggui	*Angelicae Sinensis* Radix	60	Ferulic acid	HPLC
Yuanzhi	*Polygalae Radix*	80	Ppolygalaxanthone III, 3’,6‐Disinapoylsucrose	HPLC
Dazao	*Jujubae Fructus*	60	Betulinic acid, oleanolic acid	TLC
Shichangpu	*Acorus tatarinowii* Schott	80	Unidentified volatile oil	distillation
Jiangbanxia	*Pinelliae Rhizoma* Praeparatum cum *Zingbere et Alumine*	60	None	
Baizhu	*Atractylodis Macrocephalae* Rhizoma	60	Atractylo	TLC
Fuxiaomai	Blighted wheat	200	None	
Baihe	*Lilii Bulbus*	200	Unidentified polysaccharide	Colorimetry
Dannanxing	*Arisaema* Cum Bile	80	None	
Yujin	*Curcuma wenyujin*	80	None	
Longchi	*Dens Draconis*	200	None	
Suanzaoren	*Ziziphi Spinosae* Semen	100	Jujuboside A and spinosin	HPLC
Fuling	*Poria*	100	None	
*Jieyu Anshen* Granule		1580	Geniposide	HPLC

As seen in Table 1, Pharmacopeia used a conventional HPLC method to determine geniposide, to assess the quality of whole *Jieyu Anshen* Granule. However, this method only characterizes the presence of Zhizi, as geniposide is specific to Zhizi and serves as the quality‐marker (Q‐marker) for Zhizi alone. This may lead to adulteration of other herbal medicines. For example, if Danggui is absent or replaced by wood powder in the Granule, such adulteration cannot be recognized by current Pharmacopeia quality assessment strategy, because Danggui's Q‐marker (ferulic acid) is not included in the quality‐assessment strategy.

In order to establish a reliable and effective quality assessment strategy for *Jieyu Anshen* Granule, the present study meticulously selected a set of authentic standards. These standards were then subjected to analysis using the emerging ultra‐high‐performance liquid chromatography‐Quadrupole‐Orbitrap‐tandem mass spectrometry (UHPLC−Q‐Orbitrap‐MS/MS), to create a specialized library. It's worth noting that all of the standards used were derived from traditional Chinese herbal medicines, making the library highly specialized in the field of TCM.

Using the same conditions as the TCM‐specific library, *Jieyu Anshen* Granule was also analyzed using UHPLC−Q‐Orbitrap‐MS/MS technology. This analysis allowed for the simultaneous and putative identification of various compounds in *Jieyu Anshen* Granule through library comparison. Thereafter, some identified compounds were proposed as new and additional Q‐markers for the Pharmacopeia. The integration of these proposed Q‐markers with the aforementioned UHPLC−Q‐Orbitrap‐MS/MS analysis would provide an effective and reliable strategy for assessing the quality of *Jieyu Anshen* Granule for Pharmacopeia according to Pharmacopeia standards. owing to its high accuracy, UHPLC−Q‐orbitrap MS/MS analysis is believed to unveil previously unexpected compounds.

In addition, computational chemistry approach would also be introduced in the study, to calculate the optimized conformation and energy gap of proposed Q‐markers. All these experimental and computational approaches would not only offer new information regarding the chemistry of Jieyu *Anshen* Granule, but also enhance the reliability of proposal of Q‐markers.

## Materials and Methods

### Medicine and Chemicals

Jieyu Anshen Granule was purchased from Xinhui Pharmaceutical Co., LTD (20220322, Jilin, China). Methanol and water were of mass spectra purity grade. All other reagents used in this study were purchased as analytical grade from the Guangzhou Chemical Reagent Factory (Guangzhou, China).

### Authentic Standards

5‐Caffeoylquinicacid (Cas. 906–33‐2, C_16_H_18_O_9_, M.W. 354.309, 98 %), naringenin‐7‐O‐β‐D‐glucoside (Cas. 529–55‐5, C_21_H_22_O_10_, M.W. 434.393, 98 %), isoviolanthin (Cas. 40788–84‐9, C_27_H_30_O_14_, M.W. 578.519, 98 %), calycosin (Cas. 20575–57‐9, C_16_H_12_O_5_, M.W. 284.263, 98 %), isoliquiritigenin (Cas. 961–29‐5, C_15_H_12_O_4_, M.W. 256.253, 98 %), formononetin (Cas. 485–72‐3, C_16_H_12_O_4_, M.W. 268.264, 98 %), jervine (Cas. 469–59‐0, C_27_H_39_NO_3_, M.W. 425.603, 98 %), and lancerin (Cas. 81991–99‐3, C_19_H_18_O_10_, M.W. 406.34, 98 %) were obtained from Biopurify Phytochemicals, Ltd. (Chengdu, China). 3,3’,4’,5,6,7,8‐Heptamethoxyflavone (Cas. 1178–24‐1, C_22_H_24_O_9_, M.W. 432.421, 98 %), vicenin‐2 (Cas. 23666–13‐9, C_27_H_30_O_15_, M.W. 594.518, 98 %), and schaftoside Cas. 51938–32‐0, C_26_H_28_O_14_, M.W. 564.49, 98 %) were purchased from Sichuan Weikeqi Biological Technology Co., Ltd. (Chengdu, China). Cyclocommunol (Cas. 145643–96‐5, C_20_H_16_O_6_, M.W. 352.337, 98 %) was purchased from BioBioPha Co., Ltd. (Kunming, China). Tangeretin (Cas.481‐53‐8, C_20_H_20_O_7_, M.W. 372.369, 98 %), ferulic acid (Cas. 1135–24‐6, C_10_H_10_O_4_, M.W. 194.184, 98 %), rutin (Cas. 153–18‐4, C_27_H_30_O_16_, M.W. 610.518, 98 %), chrysoeriol (Cas. 491–71‐4, C_16_H_12_O_6_, M.W. 300.263, 98 %), 7‐O‐methylmangiferin (Cas. 31002–12‐7, C_20_H_20_O_11_, M.W. 436.366, 98 %), swertisin (Cas. 6991–10‐2, C_22_H_22_O_10_, M.W. 462.404, 98 %), hypericin (Cas. 548–04‐9, C_30_H_16_O_8,_ M.W. 504.45, 98 %), 1,2,3,7‐tetramethoxyxanthone (Cas. 22804–52‐0, C_17_H_16_O_6_, M.W. 316.3, 98 %), 7,4’‐dihydroxyflavone (Cas. 2196–14‐7, C_15_H_10_O_4_, M.W. 254.238, 98 %), and naringenin (Cas. 480–41‐1, C_15_H_12_O_5_, M.W. 272.253, 98 %) were obtained from Chengdu Alfa Biotech. Ltd. (Chengdu, China). 5‐Hydroxyflavone (Cas. 491–78‐1, C_15_H_10_O_3_, M.W. 238.238, 98 %), sucrose (Cas. 57–50‐1, C_12_H_22_O_11,_ M.W. 342.30, 98 %), ethyl stearate (Cas.111‐61‐5, C_20_H_40_O_2_, M.W. 312.53, 98 %), and (+)‐4‐cholesten‐3‐one (Cas. 601–57‐0, C_27_H_44_O, M.W. 384.638, 98 %) were from TCI Chemical Co. (Shanghai, China). trans‐Cinnamic acid (Cas. 140–10‐3, C_9_H_8_O_2_, M.W. 148.159, 98 %) and L‐proline (Cas. 147–85‐3, C_5_H_9_NO_2_, M.W. 115, 98 %) was from J&K Scientific Co., Ltd. (Beijing, China). Glycyrrhizic acid (Cas. 1405–86‐3, C_42_H_62_O_16_, M.W. 822.932, 98 %), liquiritigenin (Cas. 578–86‐9, C_15_H_12_O_4_, M.W. 256.257, 98 %), liquiritin (Cas. 551–15‐5, C_21_H_22_O_9_, M.W. 418.398, 98 %), isoliquiritin (Cas. 5041–81‐6, C_21_H_22_O_9_, M.W. 418.398, 98 %), calycosin‐7‐O‐β‐D‐glucoside (Cas. 20633–67‐4, C_22_H_22_O_10_, M.W. 446.408, 98 %), polygalaxanthone III (Cas. 162857–78‐5, C_25_H_28_O_15_, M.W. 568.484, 98 %), liquiritin apioside (Cas. 74639–14‐8, C_26_H_30_O_13_, M.W. 550.513, 98 %), 18β‐glycyrrhetinic acid (Cas. 471–53‐4, C_30_H_46_O_4_, M.W. 470.694, 98 %), citric acid (Cas. 77–92‐9, C_6_H_8_O_7_, M.W. 192.123, 98 %), ligustilide (Cas. 81944–09‐4, C_12_H_14_O_2_, M.W. 190.238, 98 %), alantolactone (Cas. 546–43‐0, C_15_H_20_O_2_, M.W. 232.323, 98 %), matrine (Cas. 519–02‐8, C_15_H_24_N_2_O, M.W. 248.37, 98 %), betaine (Cas. 107–43‐7, C_5_H_11_NO_2_, M.W. 117.148, 98 %), saikosaponin A (Cas. 20736–09‐8, C_42_H_68_O_13_, M.W. 780.982, 98 %), and saikosaponin D (Cas.20874‐52‐6 C_42_H_68_O_13_, M.W. 780.982, 98 %) were from Shaanxi Herbest Co., Ltd. (Baoji, China). Isoferulic acid (Cas. 537–73‐5, C_10_H_10_O_4_, M.W. 194.184, 98 %) and geniposide (Cas. 24512–63‐8, C_17_H_24_O_10_, M.W. 388.366, 98 %) were from Shanghai Yuanye Biotechnology Co., Ltd. (Shanghai, China). D‐gluconic acid (Cas. 526–95‐4, C_6_H_12_O_7_, M.W. 196.155, 98 %) was from Sigma‐Aldrich Co., Ltd. (Shanghai, China). Caffeine (Cas. 58–08‐2, C_8_H_10_N_4_O_2_, M.W. 194.191, 98 %) was prepared by our laboratory.^[4]^

### Preparation of Authentic Standard Solution and Sample Solution

#### Preparation of Authentic Standard Solution

All authentic standards listed in 2.2 Section were individually dissolved in methanol at 30 μg/mL concentration. The solutions were individually transferred into flask and subsequently filtered through 0.45 μm membrane. The filtrate was kept at 2–6 °C for the further analysis.^[5]^

### Preparation of Sample Solution

**Figure 2:**
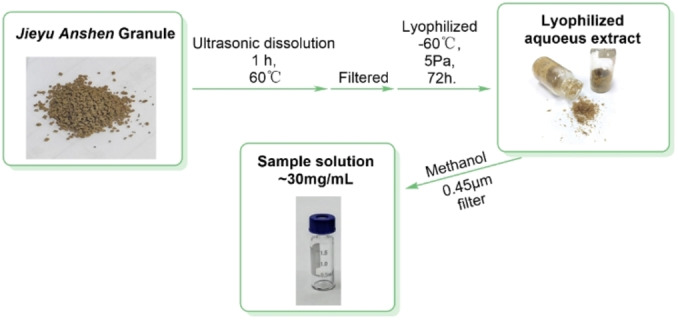
The flowchart of sample solution preparation of lyophilized aqueous extract from *Jieyu Anshen* Granule.

To avoid the possible insoluble impurity and solvent effect,^[6]^ Jieyu Anshen Granule was treated by the previous method.^[7]^ Through the treatment, the lyophilized powder of Jieyu Anshen Granule was prepared and then re‐dissolved in methanol to obtain the sample solution (30 mg/mL, Figure 2).

### UHPLC‐Q‐Exactive Orbitrap‐MS/MS Analysis

The analysis of Jieyu Anshen Granule was conducted using a UHPLC system coupling with Q‐Exactive Orbitrap mass spectrometer (Thermo Fisher Scientific, Waltham, MA, USA) equipped with a heated electrospray ionization (HESI) probe. The sample solution (at 30 mg/mL) was separated on an Accucore RP‐MS LC C_18_ column (100 mm×2.1 mm, 2.6 μm, Thermo Fisher) by binary mobile phase at 0.4 mL/min flow rate.

The binary mobile phase however consisted of mobile phase A (water, 0.1 % v/v formic acid) and mobile phase B (methanol). The linear gradient elution procedure could be described as the following: 0.0–5.0 min, 10 % B; 5.0–14.5 min, 10 % B→100 % B; 14.5–16.0 min, 100 % B; 16.0–20.0 min, 10 % B. The column chamber and sample tray were held at 40 °C and 4 °C, respectively. The electrospray ionization (ESI) parameters were set as follows: spray voltage, 4.5 kV under negative mode; sheath gas (N_2_) flow rate, 40 arbitrary units; auxiliary gas (N_2_) flow rate, 10 arbitrary units; capillary temperature, 450 °C; resolution, Ms full scan 70,000 full width at half maximum (FWHIM), MS/MS scan 17.500 FWHIM; AGC target, 2×10^5^; Stepped normalized collision energy: 20, 50, and 90; scan range, *m/z* 100–1200. An external calibration for mass accuracy was carried out before the analysis according to the manufacturer's guidelines.

### Putative Identification Using Software and MS Spectra Elucidation

The main operations regarding injection, data acquisition, and analysis were controlled by Xcalibur 4.1 package. The package comprised a TraceFinder General Quan (Thermo Fisher Scientific Inc., Waltham, MA, USA), and was equipped in the UHPLC−Q‐Exactive Orbitrap‐MS/MS apparatus. Before data acquisition, the background sign was subtracted using blank solvent. The acquired data were then exported to TraceFinder General Quan for *m/z* extraction, to form the corresponding MS spectra. Herein the processing parameters were list as follows: mass range: 100–1200 Da; mass tolerance: 5 ppm; S/N threshold: 5; isotopic pattern fit threshold: 90 %.

Subsequently, the MS spectra and corresponding top 5 secondary spectrum were screened using the Xcalibur 4.1. Through comparing with authentic standards in 4 parameters (retention time, molecular ion peak, MS/MS profile, and characteristic fragments), the compounds were putatively identified by manual.^[8]^

### Computational Chemistry Approach

All computational calculation was conducted using the Gaussian 16 software. The conformational optimization, energy optimization, and dipole moment were calculated at (U)B3LYP−D3(BJ)/6‐31+G(d,p).[^9^, ^10^, ^11^] The conformational optimization was identified by absence of imaginary frequencies. The optimized conformation was viewed and then exported via Gaussian View 6.1.1 software.^[12]^ The energy optimization included single point energy (SPE) optimization and HOMO→LUMO (from highest occupied molecular orbital to lowest unoccupied molecular orbital) energy gap optimization. Six calculated compounds were geniposide (**10**), polygalaxanthone III (**19**)**, l**igustilide (**35**), glycyrrhizic acid (**38**), saikosaponin A (**42**), and saikosaponin D (**43**). Gaussian 16 and Gaussian View 6.1.1 were manufactured by Gaussian, Inc. (Wallingford, CT, USA).

### Statistical Analysis

Each experiment of quantitative assessment was performed in triplicate. Data were shown as the means±standard deviations (SD) from three independent measurements.^[13]^

## Results

### UHPLC‐Q‐Exactive‐Orbitrap‐MS/MS Identification

**Figure 3:**
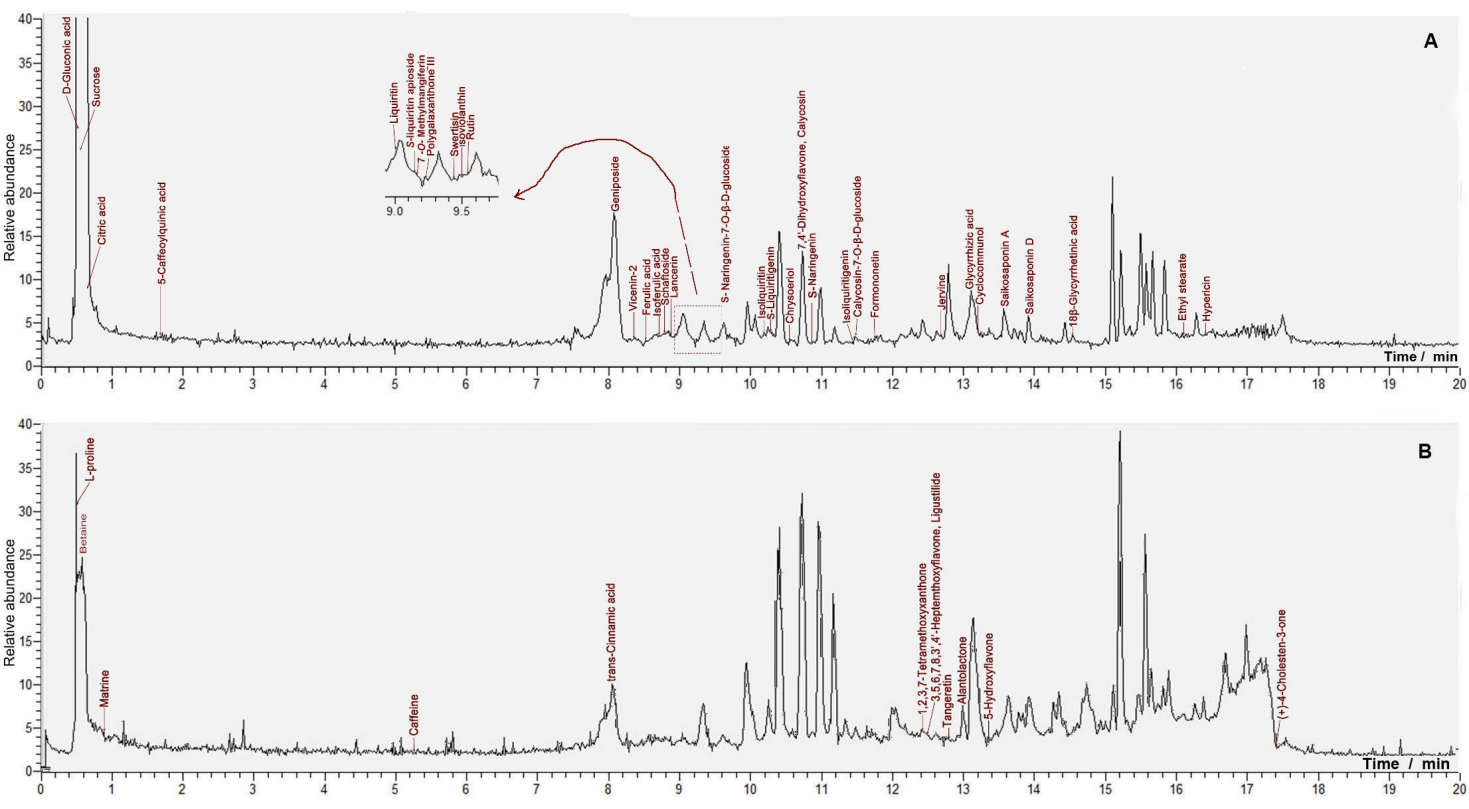
The total ion current (TIC) chromatogram of Jieyu Anshen Granule in the UHPLC−Q‐Exactive‐Orbitrap‐MS/MS analysis. (A) The negative ion mode; (B) the positive ion mode. The positive ion mode was supplement of negative ion mode.

**Figure 4:**
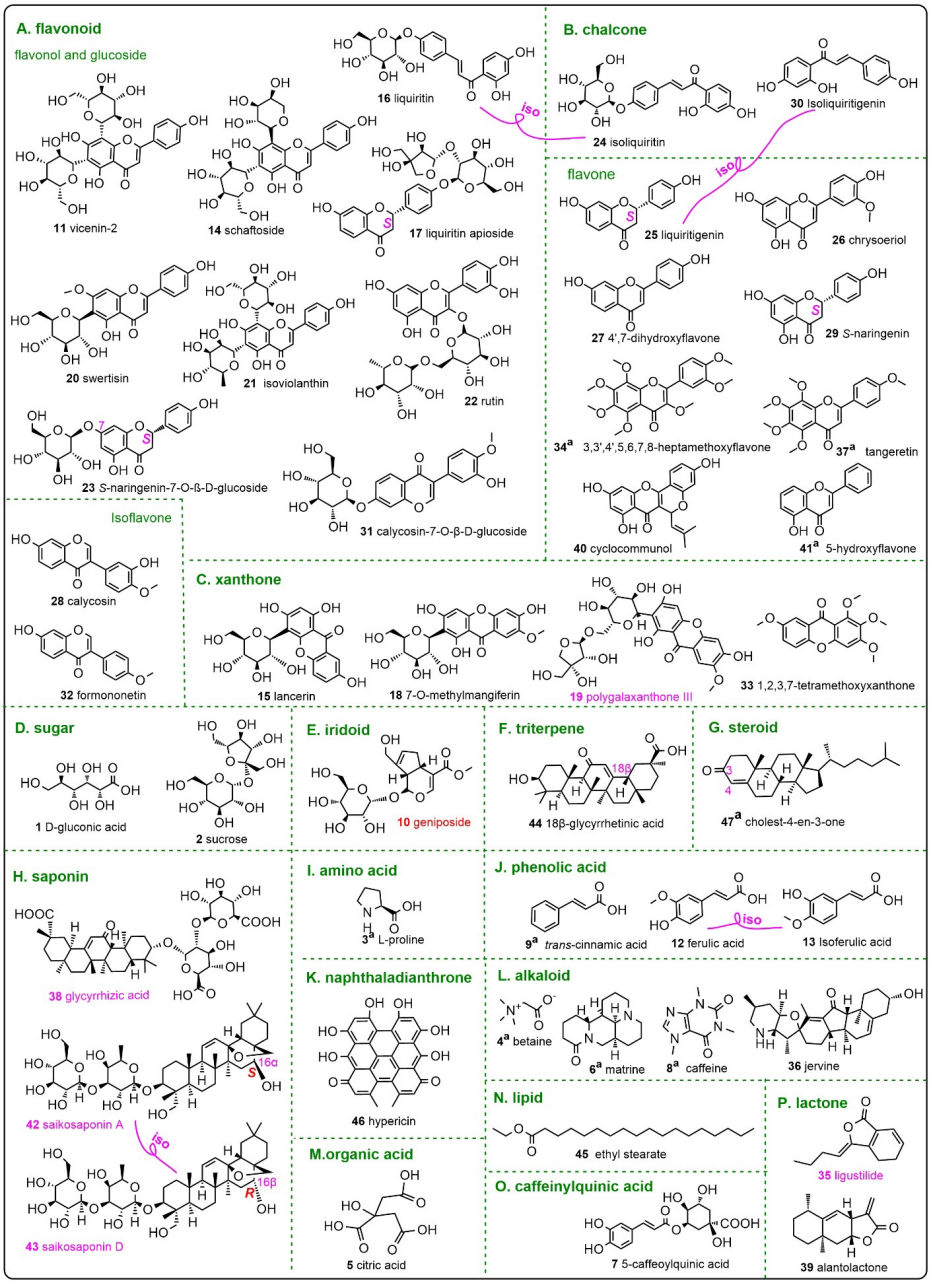
The structures and configurations of 47 identified compounds from *Jieyu Anshen* Granule. The red indicates the old Q‐marker, while the purple indicates new Q‐markers. The wave line in 35 indicated the uncertain stereo‐configuration. The symbol “a” means positive ion model. Considering the layout space, the TIC diagram under positive ion model was not shown in the main text. Knot line with “iso” connected two isomers.

**Figure 5:**
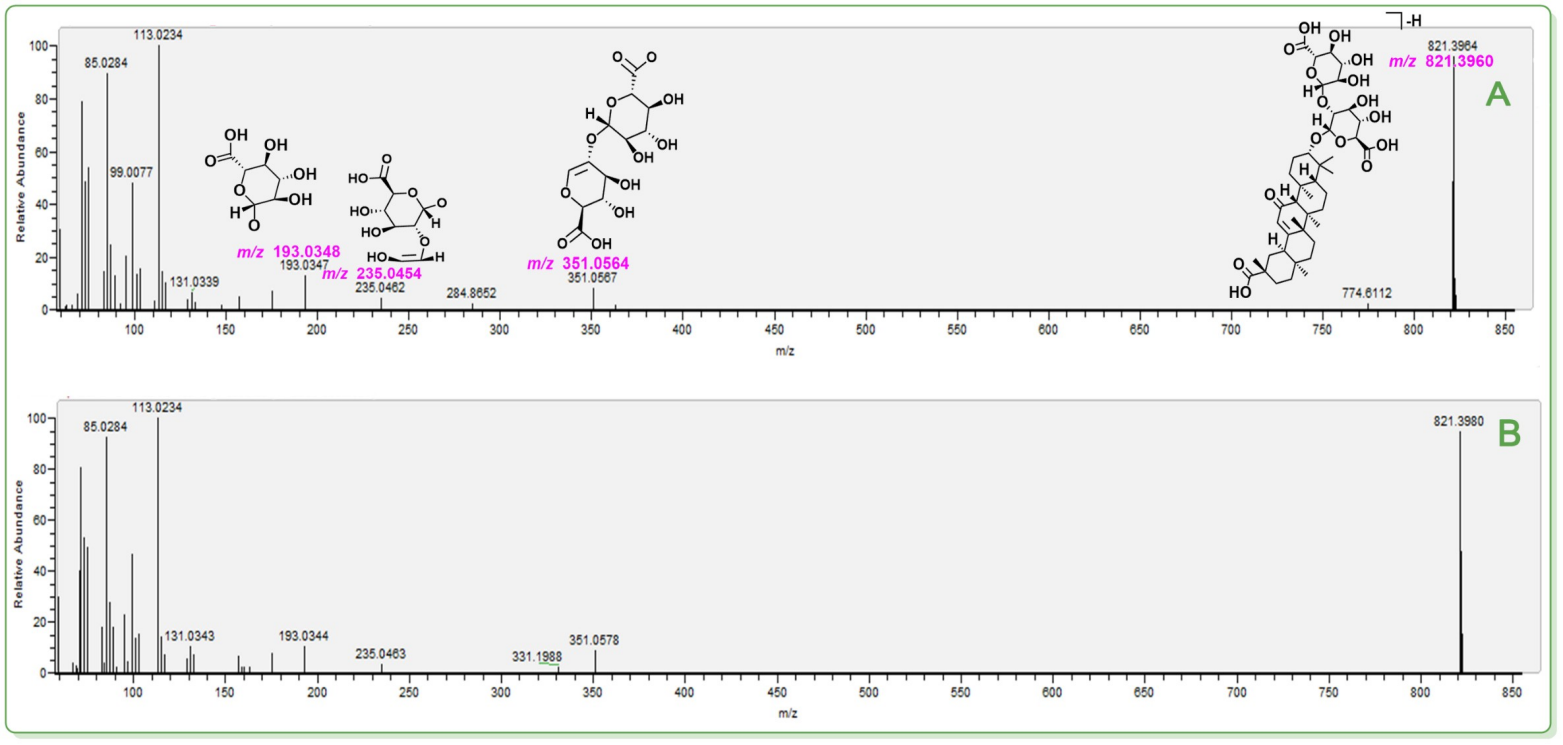
The putative identification of glycyrrhizic acid (**38**). (**A**) MS/MS spectra and the relevant elucidation of standard glycyrrhizic acid; (**B**) MS/MS spectra of the peak (R.T. 13.11). The *m*/*z* values in purple indicated the calculated ones. The relative standard deviation (RSD) values of fragments between calculated *m*/*z* values and experimental *m*/*z* values varied from 4.87×10^−7^–3.40×10^−6^. The MS/MS spectra of two corresponding standards are detailed in **Suppl. 43**.

**Figure 6:**
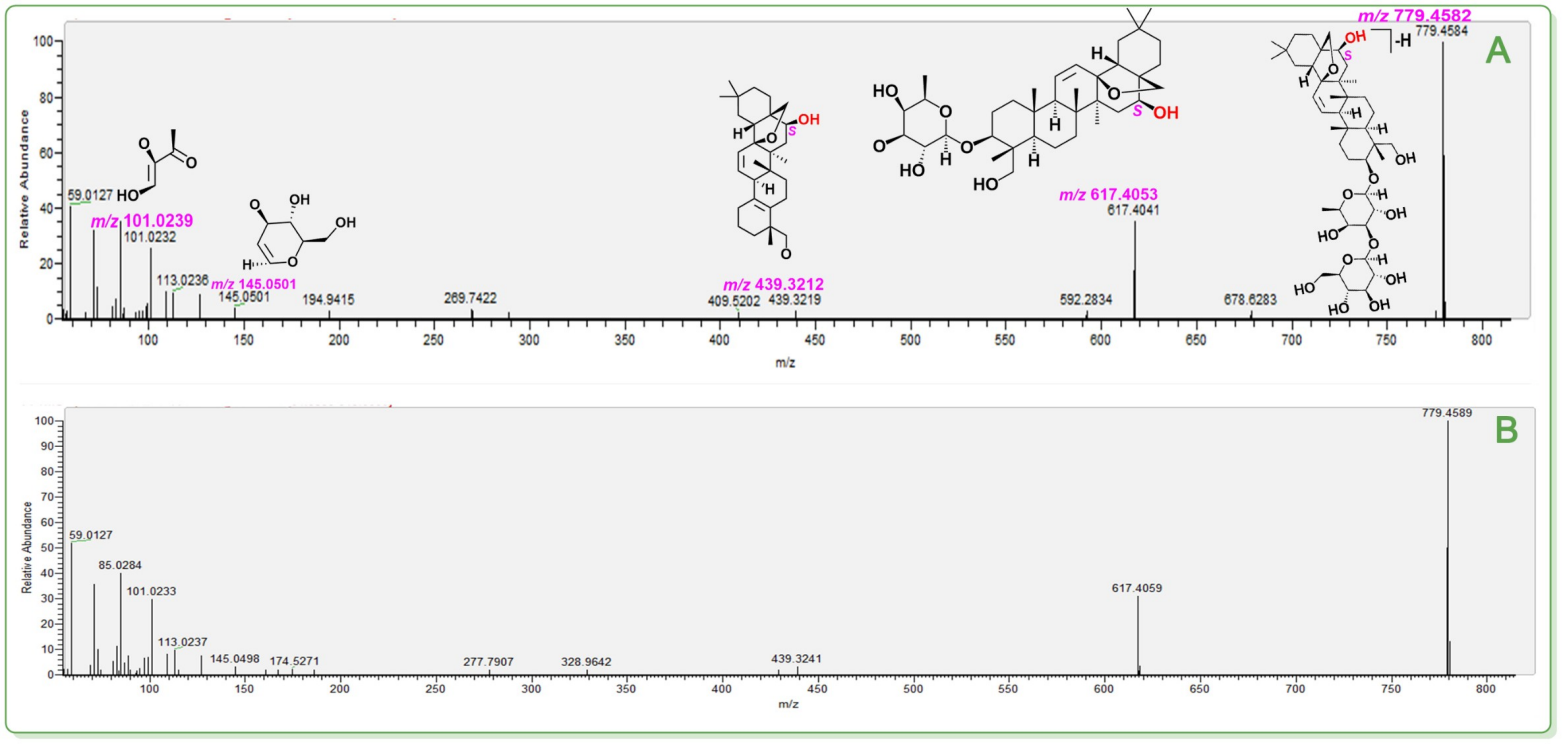
The putative identification of saikosaponin A (**42**). (**A**) MS/MS spectra and the relevant elucidation of standard saikosaponin A; (**B**) MS/MS spectra of the peak (R.T. 13.59). The *m*/*z* values in purple indicated the calculated ones. The relative standard deviation (RSD) values of fragments between calculated *m*/*z* values and experimental *m*/*z* values varied from 0–6.93×10^−6^. The MS/MS spectra of two corresponding standards are detailed in **Suppl. 43**.

**Figure 7:**
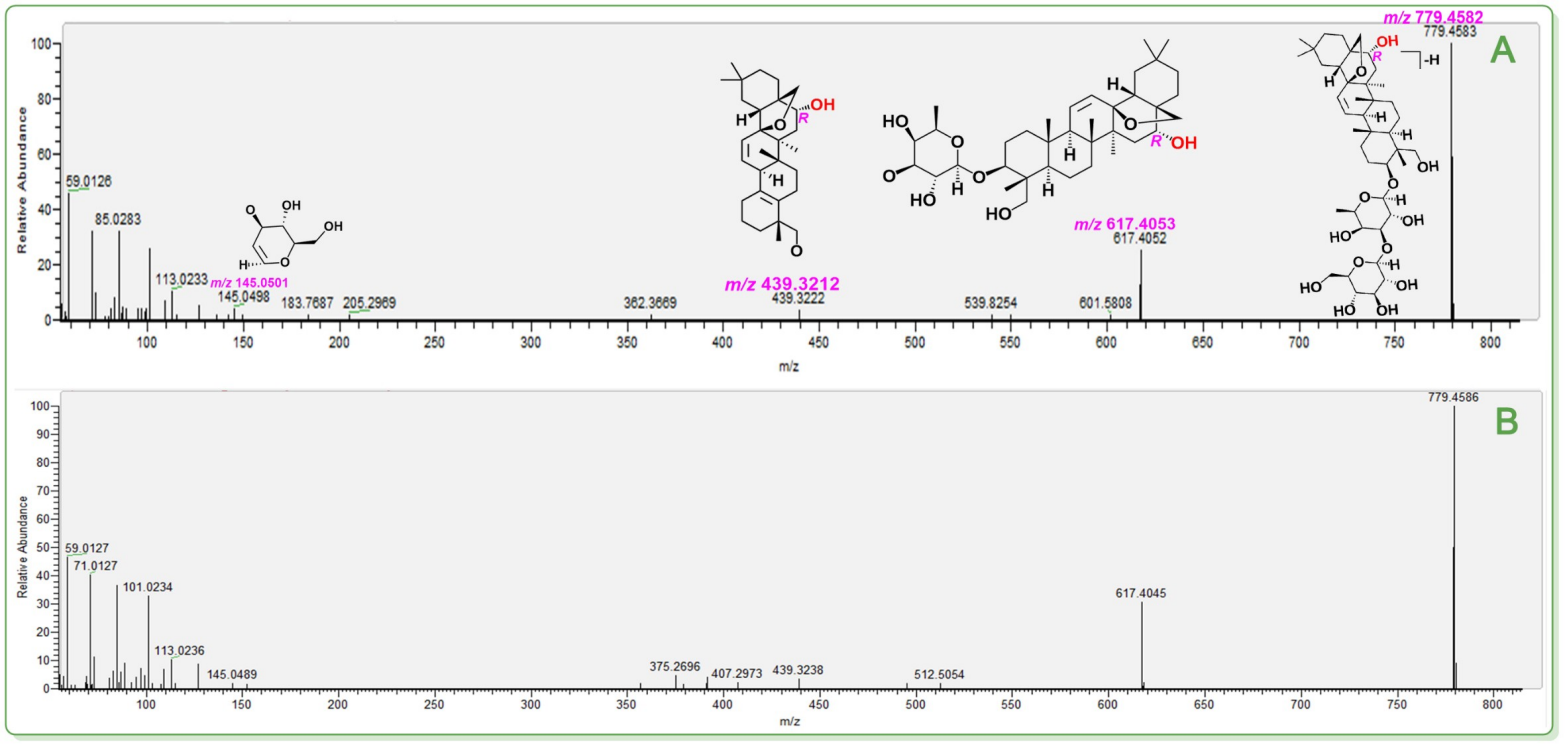
The putative identification of saikosaponin D (**43**). (**A**) MS/MS spectra and the relevant elucidation of standard saikosaponin D; (**B**) MS/MS spectra of the peak (R.T. 13.89). The *m*/*z* values in purple indicated the calculated ones. The *m*/*z* values in blue are the calculated ones. The relative standard deviation (RSD) values of fragments between calculated *m*/*z* values and experimental *m*/*z* values varied from 1.28×10^−7^–2.28×10^−6^. The MS/MS spectra of two corresponding standards are detailed in **Suppl. 43**.

**Figure 8:**
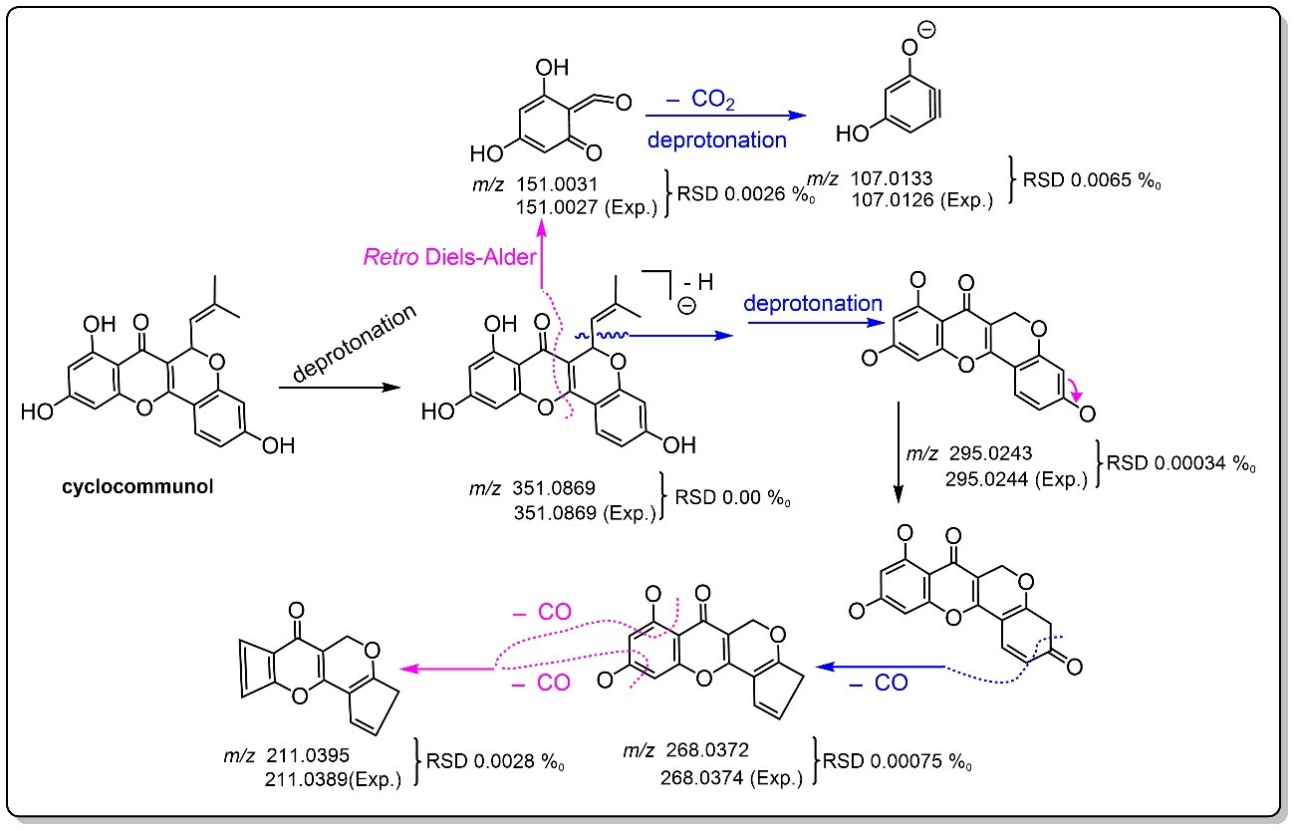
The MS fragmenting elucidation of cyclocommunol **(40)**.

The present study analyzed the sample solution of lyophilized aqueous extract from *Jieyu Anshen* Granule using UHPLC−Q‐Orbitrap MS/MS technology to obtain its total ion current (TIC) diagram (Figure 3). Additionally, the molecular formula, retention time values, and fragment *m*/*z* values were also extracted (Table 2). It was, however, necessary to compare this information with authentic standards in the library in order to identify 47 compounds (**1–47**)^[10]^ (Figure 4). Considering the layout space, the MS spectra, along with the fragmenting elucidations of all compounds, were deposited in the Suppls. 1–47. Especially, the MS spectra along with the fragmenting elucidations of three saponins **(38, 42**, and **43**) were listed in Figures 5, 6, 7 and the MS fragmenting elucidation of cyclocommunol **(40**) is illustrated in Figure 8.

**Table 2:** The main experimental results of 47 putatively identified compounds (**1–47**) in *Jieyu Anshen* Granule.

	Name	Formula	(M−H)/(M+H) *m*/*z*	R.T. min	Diagnostic fragment *m*/*z*	Plant sources
**1**	D‐Gluconic acid	C_6_H_12_O_7_	195.0505	0.51	177.0395, 159.0288, 129.0183	Baihe^[15]^
**2**	Sucrose	C_12_H_22_O_11_	341.1084	0.54	179.0553, 113.0233, 101.0233	Baihe^[15]^
**3^A^**	L‐proline	C_5_H_9_NO_2_	116.0712	0.54	70.0657, 68.0501	Danggui,^[16]^ Suanzaoren^[17]^
**4^A^**	Betaine	C_5_H_11_NO_2_	118.0868	0.55	74.0607, 59.0736, 58.0658	
**5**	Citric Acid	C_6_H_8_O_7_	191.0192	0.61	173.0083, 129.0183, 122.3034, 111.0077,	Danggui^[16]^
**6^A^**	Matrine	C_15_H_24_N_2_O	249.1967	0.87	247.1796, 218.1524, 176.1064, 148.1117	
**7**	5‐Caffeoylquinic acid	C_16_H_18_O_9_	353.0881	1.65	191.0557, 179.0341, 135.0443	Dannanxing[^18^, ^19^]
**8^A^**	Caffeine	C_8_H_10_N_4_O_2_	195.0871	5.33	163.0385, 138.0658, 123.0424, 116.9859, 110.0714	Dannanxing^[20]^
**9^A^**	*trans*‐Cinnamic acid	C_9_H_8_O_2_	149.0593	8.05	149.0593, 121.0646, 107.0490, 103.0543	Yuanzhi^[21]^
**10**	Geniposide	C_17_H_24_O_10_	387.1277	8.06	355.1029, 225.0767, 207.0661, 147.0442, 123.0441,	Zhizi^[22]^
**11**	Vicenin‐2	C_27_H_30_O_15_	593.1514	8.37	473.1100, 353.0649, 117.0336	Zhigancao^[23]^ Suanzaoren[^24^, ^25^]
**12**	Ferulic acid	C_10_H_10_O_4_	193.0502	8.52	178.0263, 149.0595, 137.0235	Baizhu,^[26]^ Danggui^[28]^ Dannanxing,^[27]^ Shichangpu^[29]^
**13**	Isoferulic acid	C_10_H_10_O_4_	193.0507	8.69	178.0263, 137.0234, 134.0365	
**14**	Schaftoside	C_26_H_28_O_14_	563.1412	8.79	473.1113, 383.0777, 353.0669, 297.0771	Dannanxing,[^30^, ^31^, ^32^] Zhigancao^[33]^
**15**	Lancerin	C_19_H_18_O_10_	405.0827	8.87	315.0518, 285.0408, 257.0455	Yuanzhi^[34]^
**16**	Liquiritin	C_21_H_22_O_9_	417.1186	9.01	255.0661, 135.0078, 119.0492	Zhigancao^[2]^
**17**	Liquiritin apioside	C_26_H_30_O_13_	549.1608	9.16	255.0660, 1135.0078, 119.0492	Zhigancao^[35]^
**18**	7‐*O*‐Methylmangiferin	C_20_H_20_O_11_	435.0932	9.17	345.0600, 315.0509, 272.0328, 243.0298, 215.0346	Yuanzhi^[34]^
**19**	Polygalaxanthone III	C_25_H_28_O_15_	567.1350	9.22	345.0612, 272.0327, 171.0445	Yuanzhi^[34]^
**20**	Swertisin	C_22_H_22_O_10_	445.1135	9.44	297.0403, 282.0538, 178.9982, 117.0336	Dazao,^[36]^ Suanzaoren^[37]^
**21**	Isoviolanthin	C_27_H_30_O_14_	577.1559	9.5	383.0772, 353.0666, 297.0775, 117.0336	Zhigancao^[38]^
**22**	Rutin	C_27_H_30_O_16_	609.1485	9.53	300.0280, 271.0257, 243.0291	Baihe,^[39]^ Chaihu,^[40]^ Dazao,^[36]^ Zhigancao,^[33]^ Suanzaoren,^[24]^ Zhizi^[41]^
**23**	Naringenin‐7‐*O*‐β‐D‐glucoside	C_21_H_22_O_10_	433.1143	9.63	271.0617, 151.0029, 119.0492	
**24**	Isoliquiritin	C_21_H_22_O_9_	417.12	10.18	297.0759, 255.0661, 135.0078	Zhigancao[^33^, ^42^]
**25**	Liquiritigenin	C_15_H_12_O_4_	255.06	10.3	135.0077, 119.0492, 117.0337	Zhigancao[^33^, ^42^]
**26**	Chrysoeriol	C_16_H_12_O_6_	299.0561	10.56	284.0330, 256.0378, 183.0443	Zhigancao^[33]^
**27**	7,4’‐Dihydroxyflavone	C_15_H_10_O_4_	253.0505	10.7	223.0396, 135.0081, 117.0336	
**28**	Calycosin	C_16_H_12_O_5_	283.0609	10.7	268.0379, 239.0355, 211.0399, 195.0450, 183.0450	Zhigancao^[38]^
**29**	Naringenin	C_15_H_12_O_5_	271.0613	10.87	151.0029, 119.0492, 117.0334, 107.0127	Zhigancao,^[43]^ Suanzaoren^[44]^
**30**	Isoliquiritigenin	C_15_H_12_O_4_	255.0661	11.44	135.0078, 119.0492	Zhigancao[^33^, ^42^]
**31**	Calycosin‐7*‐O‐*β‐D‐glucoside	C_22_H_22_O_10_	445.11	11.47	401.0875, 357.0983, 225.0548, 181.0652	
**32**	Formononetin	C_16_H_12_O_4_	267.0664	11.77	252.0427, 223.0397, 195.0447, 167.0495, 132.0207	Zhigancao^[45]^
**33^A^**	1,2,3,7‐Tetramethoxyxanthone	C_17_H_16_O_6_	317.1	12.44	287.0540, 259.0593, 215.0332	Yuanzhi^[21]^
**34^A^**	3,5,6,7,8,3’,4’‐Heptemethoxyflavone	C_22_H_24_O_9_	433.1474	12.48	403.1008, 345.0583, 205.0846, 165.053	
**35^A^**	Ligustilide	C_12_H_14_O_2_	191.1	12.5	173.0959, 145.1002, 129.0697, 115.0542, 105.0700	Danggui[^16^, ^28^, ^46^, ^47^]
**36**	Jervine	C_27_H_39_NO_3_	424.2862	12.72	248.1652, 179.1073, 163.1120	
**37^A^**	Tangeretin	C_20_H_20_O_7_	373.1267	12.84	343.0801, 297.0736, 183.0284, 135.0436	
**38**	Glycyrrhizic acid	C_42_H_62_O_16_	821.3980	13.11	351.0578, 235.0463, 193.034484	Zhigancao^[48]^
**39^A^**	Alantolactone	C_15_H_20_O_2_	233.15	13.12	187.1474, 145.1008, 131.0852	
**40**	Cyclocommunol	C_20_H_16_O_6_	351.0875	13.19	151.0027, 107.0493	
**41^A^**	5‐Hydroxyflavone	C_15_H_10_O_3_	239.0695	13.39	165.0539, 139.0537, 137.0230, 103.0543	
**42**	Saikosaponin A	C_42_H_68_O_13_	779.4589	13.59	617.4059, 439.3241, 145.0498, 101.0239	Chaihu^[49]^
**43**	Saikosaponin D	C_42_H_68_O_13_	779.4586	13.89	617.4045, 439.3238, 145.0489	Chaihu^[49]^
**44**	18β‐Glycyrrhetinic acid	C_30_H_46_O_4_	469.33	14.55	425.3433, 355.2642	Zhigancao^[48]^
**45**	Ethyl Stearate	C_20_H_40_O_2_	311.2959	16.11	183.0114, 119.0492	Suanzaoren^[17]^
**46**	Hypericin	C_30_H_16_O_8_	503.08	16.42	459.0873, 405.0771, 361.0877	
**47^A^**	(+)‐4‐Cholesten‐3‐one	C_27_H_44_O	385.3448	17.39	367.3326, 123.0800, 109.0648	Dannanxing^[50]^

**Note**: The compounds with serial number “A” were identified by positive ion mode. The *m*/*z* value in bold means the characteristic fragments. The *m*/*z* values below 50 were also found by the Xcalibur 4.1 Software package, despite that the scan mode rang was set at *m*/*z* 100–1200 in the mass spectra. The table only listed the diagnostic fragment of MS/MS spectra. All Other fragments of MS/MS spectra were detailed in Suppls. 1–47. The fragmenting pathway elucidation was also shown in Suppls. 1–47.

### Results of Computational Chemistry

**Figure 9:**
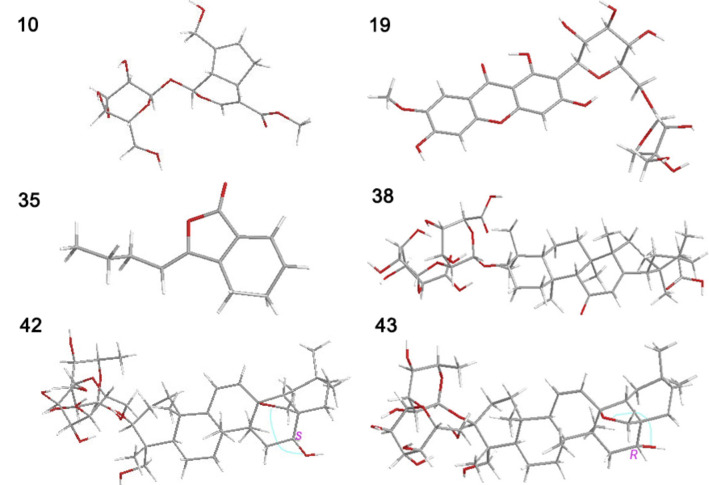
The optimized conformation of geniposide (**10**), polygalaxanthone III (**19**), ligustilide (**35**), glycyrrhizic acid (**38**), saikosaponin A (**42**), and saikosaponin D (**43**). The calculation was conducted at (U)B3LYP−D3(BJ)/6‐31+G(d,p) basis set with SMD model using the Gaussian 16 software and Gaussian View 6.1.1.

Computational chemistry was performed using Gaussian 16 software. Computational results, including conformational optimization and the calculation of various parameters, were analyzed using Gaussian View 6.1.1. The optimized conformations are presented in Figure 9, and additional parameters are listed in Table 3.

**Table 3:** The relevant parameters of geniposide (**10**), polygalaxanthone III (**19**), ligustilide (**35**), glycyrrhizic acid (**38**), saikosaponin A (**42**), and saikosaponin D (**43**).

	Q‐markers	SPE	Dipole moment	HOMO→LUMO
**10**	geniposide	−1414.4959	7.3381	0.2097
**19**	polygalaxanthone III	−2097.8782	6.8814	0.1541
**35**	ligustilide	−616.1624	5.1278	0.1402
**38**	glycyrrhizic acid	−2841.4520	9.5124	0.1822
**42**	saikosaponin A	−2619.2731	5.9957	0.2404
**43**	saikosaponin D	−2619.2722	5.9992	0.2417

RB3LYP, restricted B3LYP basis set; SPE, single point energy, Hartree unit; dipole moment value, Debye unit; HOMO→LUMO, the energy gap from highest occupied molecular orbital to lowest unoccupied molecular orbital, a.u. unit, 1 a.u.=2625.5 kJ/mol.

## Discussion

In the present study, a total of 47 compounds were identified simultaneously from *Jieyu Anshen* Granules, as shown in Figure 4. From an efficiency perspective, it presents an advantage over conventional HPLC‐UV analysis, which can only identify a limited number of compounds within the Granule.^[38]^ This, of course, can be attributed to the application of UHPLC−Q‐Orbitrap MS/MS technology itself, because similar high‐efficiency identification was also reported in the previous studies. However, these studies overlooked the distinction of isomers.^[39]^

In the study, liquiritin (**16)** and isoliquiritin (**24) contructed** a pair of structural isomers, for their structural skeletons differ. Our previous study demonstrated that the former skeleton could be synthesized from the latter skeleton with the catalysis of chalcone isomerase (CHI).^[40].^ However, two isomers have been clearly distinguished from each other in the study (Suppls. 16 and 24). Another pair of skeleton isomers (**25** and **30**) was also successfully distinguished from each other. Additionally, a pair of positional isomers (**12** and **13**) has been strictly distinguished as well (see Figure 4). The successful differentiation of these isomers was made possible by a novel method developed by our research team.^[41]^

Taken together, our study combined the TCM‐specific library with UHPLC−Q‐Orbitrap‐MS/MS analysis to achieve isomer distinction. These achievements can ultimately be attributed to the high‐accuracy MS/MS fragmenting elucidation. In Figures 5–7 (Suppls. 1–47), it is evident all compounds have been elucidated using the diagnostic MS/MS fragment based on the MS spectra principle (e. g., *Retro* Diels‐Alder fragmenting, Figure 8). The RSD values between the calculated and experimental *m*/*z* values were on the order of magnitude of 10^−6^ (e. g., 0.0000 ‰~0.0065 ‰, see Figure 8). It has been suggested that our work was highly reliable and was thus regarded as putative identification. In contrast, the previous tentative identifications were incapable of isomer distinction. For instance, three teams did not elucidate the MS/MS fragmenting, thus rendering them unable to distinguish isomers.[^42^, ^43^, ^44^]

Among these putatively identified compounds, twelve were described as unexpected, as there were no prior reports indicating their presence in the Granule, despite a systematic document retrieval effort. These twelve compounds included betaine (**4)**, matrine (**6)**, naringenin‐7*‐O‐*β‐D‐glucoside (**23)**, 7,4’‐dihydroxyflavone (**27)**, calycosin‐7*‐O‐*β‐D‐glucoside (**31)**, 3,5,6,7,8,3’,4’‐heptemethoxyflavone (**34)**, tangeretin (**37)**, jervine (**36**), alantolactone (**39**), cyclocommunol (**40**), 5‐hydroxyflavone (**41)**, and hypericin (**46**).

In particular, eight unexpected compounds (**6**, **31**, **34**, **36**, **37**, **40**, **41**, and **46**) showed low peak signs in the TIC diagram (see Figure 3). Consequently, they were marked as “unexpected and trace level”. Instances of similar unexpected and trace level compounds have been noted in prior UHPLC−Q‐Orbitrap‐MS/MS analysis.^[6]^ In contrast, four unexpected compounds (**4**, **23**, **27**, and **39**) showed strong peak signs in the TIC diagram (see Figure 3), which led to their classification as “unexpected and substantial level” compounds. Similar unexpected and substantial level instances have been recently reported in the UHPLC−Q‐Orbitrap‐MS/MS analyses of pomelo peel.^[45]^ It is worth noting that phytochemical studies related to pomelo peel were initiated in the 1980s^[46]^, predating our findings. This means that the phytochemical work is an ongoing process and our finding of twelve unexpected compounds may be omitted by previous phytochemical workers. In addition to providing guidance for future phytochemical research, the discovery of “unexpected” compounds will also shed light on the chemistry of *Jieyu Anshen* Granule or its relevant plants. In fact, two unexpected and substantial level compounds, betaine and alantolactone have been reported to possess bioactive effects similar to those found in the Granule.[^47^, ^48^]

All these expected and unexpected compounds, however, could be classified into sixteen main types, including flavonoid, xanthone, alkaloid, saponin, phenolic acid, chalcone, sugar, triterpene, steroid, lactone, iridoid, amino acid, naphthaladianthrone, organic acid, lipid, and caffeoylquinic acid (Figure 4), from a phytochemical perspective. This chemical diversity may account for the therapeutic effect of the *Jieyu Anshen* Granule as a whole.

As shown in Figure 3, iridoid geniposide (**10**) exhibited a prominent peak at a retention time of 8.06 minutes, indicating its robust detectability. In addition, it was also documented to possess a similar antidepressant pharmacological effect to *Jieyu Anshen* Granule.^[49]^ Possibly owing to these, geniposide was selected as the Pharmacopeia Q‐marker previously.^[2]^

Herein we aimed to introduce new Pharmacopeia Q‐markers, adhering to the five principles outlined by Academician Liu Changxiao.^[50]^ Aside from chemical stability, non‐industrialization principles should also be taken into account.^[51]^ The former could explain the traceability, while the latter could prevent safety tragedies. This is because that industrialized Q‐marker would be illegally added into *Jieyu Anshen* Granule, causing a tragedy similar to the Sanlu Melamine Incident in China (2008). In accordance with these principles, five compounds (**19, 35**, **38, 42**, and **43**) were proposed as potential new Q‐marker, as summarized in (see Table 4).

**Table 4:** The compliance with relevant principles of 5 new Q‐marker candidates (polygalaxanthone II **19**, ligustilide **35**, glycyrrhizic acid **38**, saikosaponin A **42**, and saikosaponin D **43**).

	19	35	38	42	43
Traceability	√; Ref.^[52]^	√; Ref.^[2]^	√; Ref.^[38]^	√; Ref.^[53]^	√; Ref.^[53]^
Testability	√; Figure 3	√; Figure 3	√; Figure 3	√; Figure 3	√; Figure 3
Specificity	√; Ref.^[2]^	√; Ref.^[2]^	√; Ref.^[2]^	√; Ref.^[2]^	√; Ref.^[2]^
Efficiency‐relevance	√; Ref.^[52]^	√; Ref.^[54]^	√; Ref.^[55]^	√; Ref.^[56]^	√; Ref.^[56]^
TCM‐relevance	√; Table 1	√; Table 1	√; Table 1	√; Table 1	√; Table 1
Chemical stability	√; Table 3	√; Table 3	√; Table 3	√; Table 3	√; Table 3
Non‐industrialization	√	√	√; Ref.^[2]^	√; Ref.^[2]^	√; Ref.^[2]^

Five new Q‐markers, in addition to the old one, have been utilized to construct a new Q‐marker system. The system coupling with the UHPLC−Q‐Orbitrap‐MS/MS analysis is poised to establish a novel quality assessment strategy. By integrating the plant resource information (Table 2 **)** and Pharmacopeia evidence,^[2]^ new quality assessment strategy was able to judge the adulteration of *Jieyu Anshen* Granule.

In accordance with Pharmacopeia and literature,[^2^, ^16^] polygalaxanthone III (**19**) serves as a key active compound, making it suitable for the characterization of individual Yuanzhi. In fact, it could be readily detected in plant extract or animal plasma.^[52]^ Therefore, if polygalaxanthone III is found to be absent in *Jieyu Anshen* Granule by the quality assessment strategy, it means that Yuanzhi is absent and there is adulteration in *Jieyu Anshen* Granule.

Correspondingly, if both saikosaponins (**42** and **43**) cannot be detected, it signifies the presence of adulteration in Chaihu. This is because that (1) **42** and **43** are two Pharmacopeia Q‐markers of single Chaihu^[2]^; and (2) Chaihu is their sole plant source (Table 2). In a similar vein, the absence of ligustilide implies a lack of Danggui or adulteration. As stated in Table 2, Danggui is the sole plant source of ligustilide in *Jieyu Anshen* Granule. Additionally, ligustilide is a distinct, abundant, and detectable compound of Danggui.^[12]^ Likewise, the absence of glycyrrhizic acid suggests adulteration of Zhigancao in *Jieyu Anshen* Granule. Geniposide (**10**), the old Q‐marker (pre‐existing Q‐marker), is also subject to this logical judgment. Once geniposide is not found using our quality assessment strategy, *Jieyu Anshen* Granule will be suggested for adulteration regarding Zhizi (Table 2). In summary, our proposed quality assessment strategy allows for the specific identification of five types of adulteration in *Jieyu Anshen* Granule. The total characterizing rate (TCR) of the new strategy was 31.25 % (5÷16), while its specific characterizing rate (SCR) was also 31.25 % (5÷16), according to our previous definition.^[51]^ Using this definition, the TCR and SCR values of the old quality assessment strategy were calculated as 6.25 % (1÷16) and 6.25 %, respectively. Now it is obvious that our proposed strategy has greatly improved the reliability of quality assessment for Pharmacopeia. On the other hand, our strategy has the potential to improve the specificity of the Pharmacopeia as well, for it can distinguish *Jieyu Anshen* Granule from three other TCM prescriptions, namely *Zhizi Jinhua* Wan, *Linglianhua* Granule, and *Zhiqin Qingre* Heji. This distinction arises because all of these prescriptions use geniposide **(10)** as the sole Q‐marker. Thus, without the improved quality assessment strategy, *Jieyu Anshen* Granule could easily be mistaken for these other prescriptions.

Finally, it is worth mentioning that, (1) two epimeric saikosaponins (**42** and **43**) exhibit high similarity to each other. However, saikosaponin A positions the −OH group on the same side of the O‐bridge, whereas saikosaponin D situates the −OH group on the opposite side of the O‐bridge (see Figure 9). The distinct arrangements have differentiated the molecular polarities, allowing for their separation through conventional C_18_ adsorption chromatography column^[57]^ (refer to Table 3).

(2) Two saikosaponins (**42** and **43**) can also be interconverted through biosynthesis,^[58]^ especially when they leaped over a low energy gap (▵E=0.004 Hartree, as depicted in Figure 9C). Therefore, only when both saikosaponins are not detected by the quality assessment strategy, the adulteration of Chaihu can be considered.

(3) As seen in Table 1, betulinic acid and oleanolic acid are defined as the Pharmacopeia Q‐marker of Dazao. However, both compounds could not be detected in *Jieyu Anshen* Granule (as shown in Figure 4). This absence may be attributed to the low proportion of Dazao in the Granule which accounts for only (3.79 %, 60÷1580). It's worth noting that our TCM‐specific library includes standards for these compounds.

(4) *Jieyu Anshen* Granule is a prescription comprised of 16 TCHMs. Each CHM is recognized for containing a significant number of compounds, and some compounds may appear in multiple TCHMs. This complexity has made the Granule an exceptionally intricate chemical system. Therefore, characterization of all 16 TCHMs using a Q‐marker system remains a challenge, despite significant advancements in the study.

(5) The study regarding the Pharmacopeia quality assessment strategy is distinct from the Pharmacopeia strategy itself, as the study lacks both administrative compulsion and legal authority. The official adoption of such a strategy depends on the deliberation of the Pharmacopeia Commission. Furthermore, the Pharmacopeia Commission's deliberations are influenced by the accumulation of research publications. The Pharmacopeia Commission would not identify a new and effective quality assessment strategy without these research publications.

## Conclusions

Through the application of UHPLC−Q‐Orbitrap‐MS/MS for putative identification, it has been confirmed that the *Jieyu Anshen* Granule contains a minimum of 47 compounds, comprising 12 unexpected compounds and 35 expected compounds. These unexpected compounds include cyclocommunol, 5‐hydroxyflavone, tangeretin, 3,5,6,7,8,3’,4’‐heptemethoxyflavone, calycosin‐7‐*O*‐β‐D‐glucoside, 7,4’‐dihydroxyflavone, naringenin‐7‐*O*‐β‐D‐glucoside, matrine, betaine, alantolactone, and hypericin. Among the expected compounds, saponin saikosaponin A and its epimer saikosaponin D can be easily separated in the C_18_ column. Saikosaponins A and D, along with glycyrrhizic acid, geniposide, ligustilide, and polygalaxanthone III can establish a novel quality assessment strategy to identify potential adulteration related to Zhizi, Chaihu, Zhigancao, Danggui, and Yuanzhi in *Jieyu Anshen* Granule. Therefore, we proposed its consideration by the Pharmacopeia Commission. Additionally, the identification of these 12 unexpected compounds will significantly benefit its future chemical or pharmacological research.

## Ethical Approval

Not applicable.

## Author Contributions

XL contributed to the project design and paper writing. JZ and XC contributed to literature review and analysis experiments. RC, SK and JZ contributed to data analyses. CL and BC contributed to computational chemistry. XZ contributed to paper revision. All authors read and approved the final manuscript.

## Conflict of Interests

The authors declare no conflict of interest.

1

## Supporting information

As a service to our authors and readers, this journal provides supporting information supplied by the authors. Such materials are peer reviewed and may be re‐organized for online delivery, but are not copy‐edited or typeset. Technical support issues arising from supporting information (other than missing files) should be addressed to the authors.

Supporting Information

## Data Availability

All the data used to support the findings of this study are available from the corresponding author upon reasonable request.
