# Supplementary material for: Putative Identification of 47 Compounds from Jieyu Anshen Granule and Proposal of Pharmacopeia Quality‐Assessment Strategy Using TCM‐Specific Library with UHPLC‐Q‐Exactive‐Orbitrap‐MS
Source: ChemistryOpen. 2024 Nov 21;14(1):e202400046. doi: 10.1002/open.202400046 (PMC11726651; doi:10.1002/open.202400046)
Supplement: Supplementary file 1 — Supporting Information [file OPEN-14-e202400046-s001.pdf]

ChemistryOpen

Supporting Information

Putative Identification of 47 Compounds from *Jieyu Anshen* Granule and Proposal of Pharmacopeia Quality-Assessment Strategy Using TCM-Specific Library with UHPLC-Q-Exactive-Orbitrap-MS

Xican Li,* Jingyuan Zeng, Rongxin Cai, Chunhou Li, Xiaoshan Chen, Ban Chen, Xiaojun Zhao, and Sunbal Khan

Suppl. 1 Identification of D-gluconic acid (CAS 526-95-4, $C_6H_{12}O_7$, M.W., 196.155).

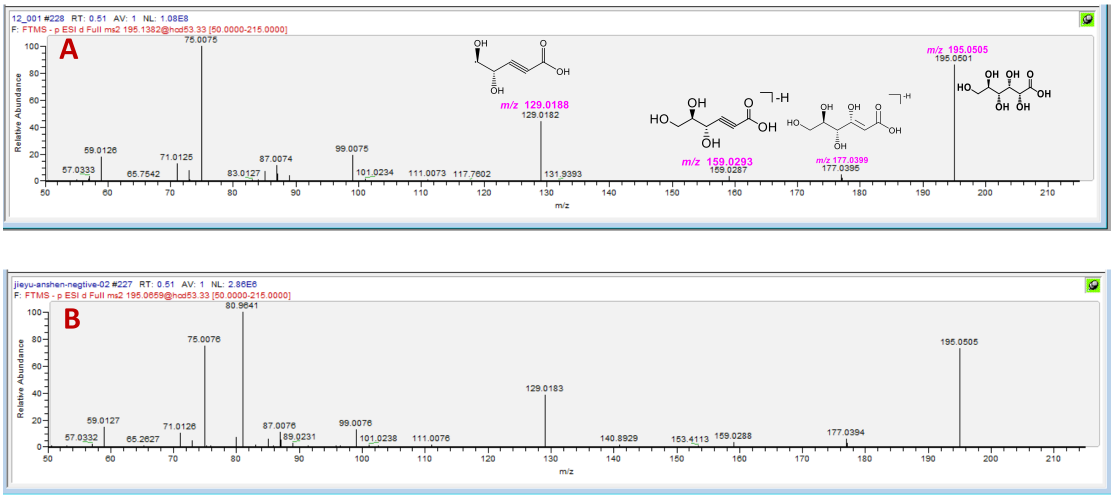

Fig. S1.1 The main results of standard D-gluconic acid (CAS 526-95-4, $C_6H_{12}O_7$) and its corresponding peak in the TIC diagram using UPLC-Q-Orbitrap-MS analysis. (**A**) The MS/MS fragments of standard D-gluconic acid. (**B**) The MS/MS spectra from chromatographic peak in R.T. 0.51 min in the *Jieyu Anshen* Granule extract.

Note: The *m/z* values in purple are the calculated ones. The *m/z* calculation was based on the relative atomic masses of C (12.0000), H (1.007825), O (15.994915), and N (14.003074)[1].

Identification: As seen in Fig. S1.1, the R.T. value, molecular ion peak, MS/MS spectra, and characteristic pears were highly similar. Thus, the chromatographic peak in R.T. 0.51 min in the *Jieyu Anshen* Granule extract was identified as D-gluconic acid (CAS 526-95-4).

References:

[1] Gross., J.H., *Mass spectrometry* Beijing: Science press, 2013. **1**.

Suppl. 2 Identification of sucrose (CAS 57-50-1, $C_{12}H_{22}O_{11}$).

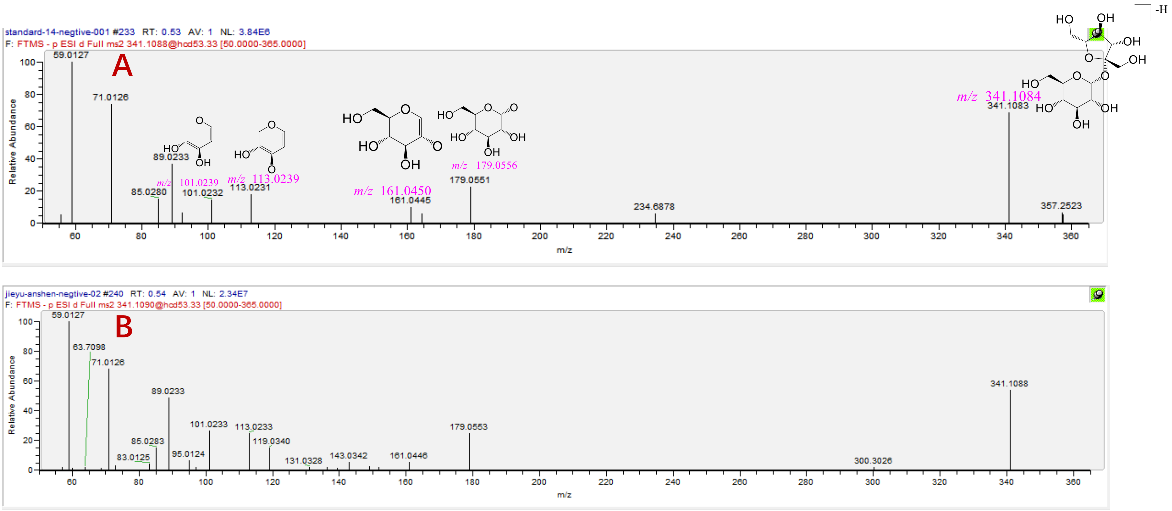

Fig. S2.1 The main results of standard Sucrose (CAS 57-50-1, $C_{12}H_{22}O_{11}$) and its corresponding peak in the TIC diagram using UPLC-Q-Orbitrap-MS analysis. (**A**) The MS/MS fragments of standard Sucrose. (**B**) The MS/MS spectra from chromatographic peak in R.T. 0.54 min in the *Jieyu Anshen* Granule extract.

Note: The *m/z* values in purple are the calculated ones. The *m/z* calculation was based on the relative atomic masses of C (12.0000), H (1.007825), O (15.994915), and N (14.003074)[1].

Identification: As seen in Fig. S2.1, the R.T. value, molecular ion peak, MS/MS spectra, and characteristic pears were highly similar. Thus, the chromatographic peak in R.T. 0.54 min in the *Jieyu Anshen* Granule extract was identified as sucrose (CAS 57-50-1, $C_{12}H_{22}O_{11}$).

References:

[1] Gross., J.H., *Mass spectrometry* Beijing: Science press, 2013. **1**.

Suppl. 3 Identification of L-proline (CAS 147-85-3, $C_5H_9NO_2$)

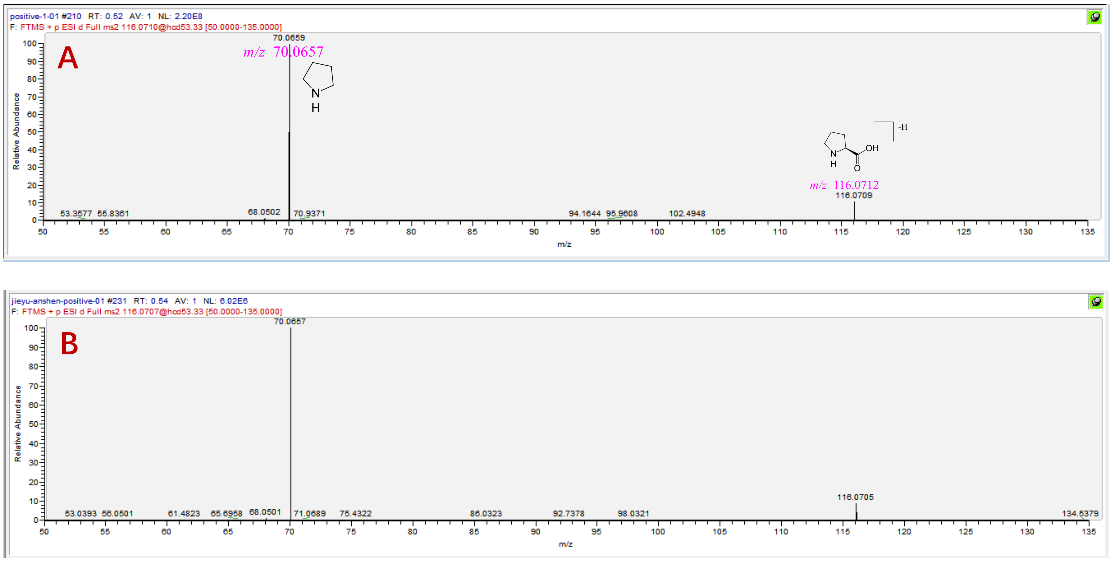

Fig. S3.1 The main results of standard L-proline (CAS 147-85-3, $C_5H_9NO_2$) and its corresponding peak in the TIC diagram using UPLC-Q-Orbitrap-MS analysis. (**A**) The MS/MS fragments of standard L-proline. (**B**) The MS/MS spectra from chromatographic peak in R.T. 0.54 min in the *Jieyu Anshen* Granule extract.

Note: The m/z values in purple are the calculated ones. The m/z calculation was based on the relative atomic masses of C (12.0000), H (1.007825), O (15.994915), and N (14.003074)[1].

Identification: As seen in Fig. S3.1, the R.T. value, molecular ion peak, MS/MS spectra, and characteristic pears were highly similar. Thus, the chromatographic peak in R.T. 0.54 min in the *Jieyu Anshen* Granule extract was identified as L-proline (CAS 147-85-3, $C_5H_9NO_2$).

References:

[1] Gross., J.H., *Mass spectrometry* Beijing: Science press, 2013. **1**.

Suppl. 4 Identification of betaine (CAS 107-43-7, $C_5H_{11}NO_2$, M.W. 117.15)

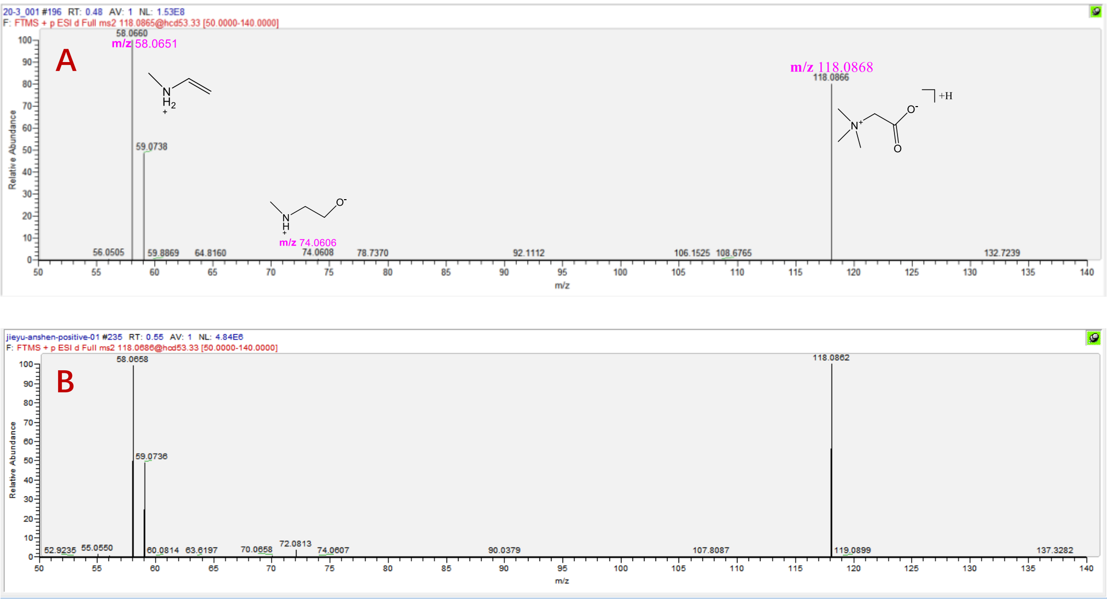

Fig. S4.1 The main results of standard betaine (CAS 107-43-7, $C_5H_{11}NO_2$) and its corresponding peak in the TIC diagram using UPLC-Q-Orbitrap-MS analysis. (**A**) The MS/MS fragments of standard Betaine. (**B**) The MS/MS spectra from chromatographic peak in R.T. 0.55 min in the *Jieyu Anshen* Granule extract.

Note: The m/z values in purple are the calculated ones. The m/z calculation was based on the relative atomic masses of C (12.0000), H (1.007825), O (15.994915), and N (14.003074)[1].

Identification: As seen in Fig. S4.1, the R.T. value, molecular ion peak, MS/MS spectra, and characteristic pears were highly similar. Thus, the chromatographic peak in R.T. 0.55 min in the *Jieyu Anshen* Granule extract was identified as Betaine (CAS 107-43-7).

References:

[1] Gross., J.H., Mass spectrometry Beijing: Science press, 2013. 1.

Suppl. 5 Identification of Citric acid (CAS 77-92-9, $C_6H_8O_7$)

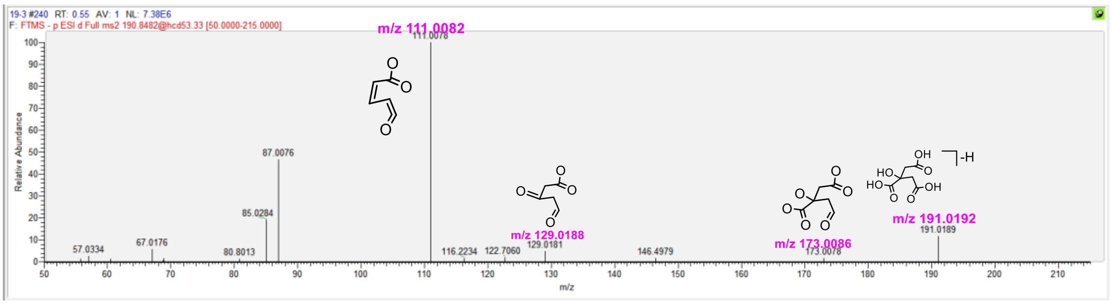

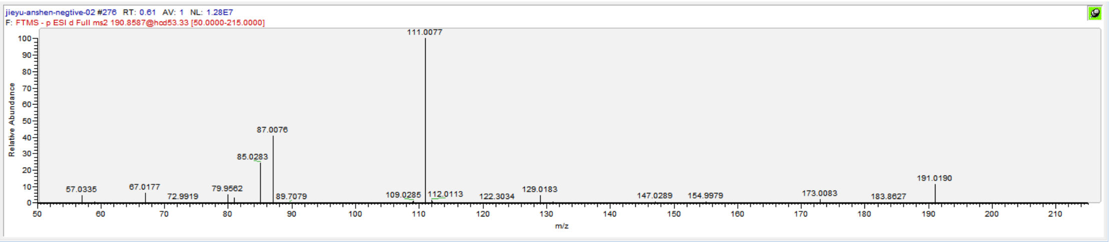

Fig. S5.1 The main results of standard citric acid (CAS 77-92-9, $C_6H_8O_7$) and its corresponding peak in the TIC diagram using UPLC-Q-Orbitrap-MS analysis. (**A**) The MS/MS fragments of standard citric acid. (**B**) The MS/MS spectra from chromatographic peak in R.T. 0.61 min in the *Jieyu Anshen* Granule extract.

Note: The m/z values in purple are the calculated ones. The m/z calculation was based on the relative atomic masses of C (12.0000), H (1.007825), O (15.994915), and N (14.003074)[1].

Identification: As seen in Fig. S5.1, the R.T. value, molecular ion peak, MS/MS spectra, and characteristic pears were highly similar. Thus, the chromatographic peak in R.T. 0.61 min in the *Jieyu Anshen* Granule extract was identified as citric acid (CAS 77-92-9, $C_6H_8O_7$).

References:

[1] Gross., J.H., Mass spectrometry Beijing: Science press, 2013. 1.

Suppl. 6 Identification of matrine (CAS 519-02-8, $C_{15}H_{24}N_2O$)

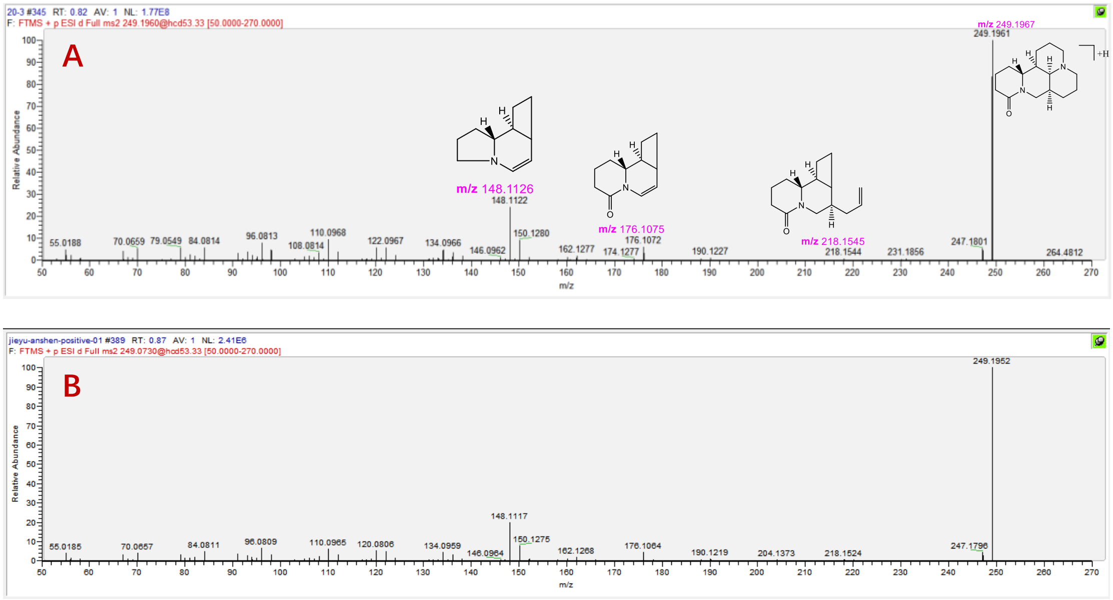

Fig. S6.1 The main results of standard matrine (CAS 519-02-8, $C_{15}H_{24}N_2O$) and its corresponding peak in the TIC diagram using UPLC-Q-Orbitrap-MS analysis. (**A**) The MS/MS fragments of standard Matrine. (**B**) The MS/MS spectra from chromatographic peak in R.T. 0.87 min in the *Jieyu Anshen* Granule extract.

Note: The m/z values in purple are the calculated ones. The m/z calculation was based on the relative atomic masses of C (12.0000), H (1.007825), O (15.994915), and N (14.003074)[1].

Identification: As seen in Fig. S6.1, the R.T. value, molecular ion peak, MS/MS spectra, and characteristic pears were highly similar. Thus, the chromatographic peak in R.T. 0.87 min in the *Jieyu Anshen* Granule extract was identified as Matrine (CAS 519-02-8, $C_{15}H_{24}N_2O$)

References:

[1] Gross., J.H., Mass spectrometry Beijing: Science press, 2013. 1.

Suppl. 7 Identification of 5-caffeoylquinicacid (CAS 906-33-2, $C_{16}H_{18}O_9$, M.W., 354.309).

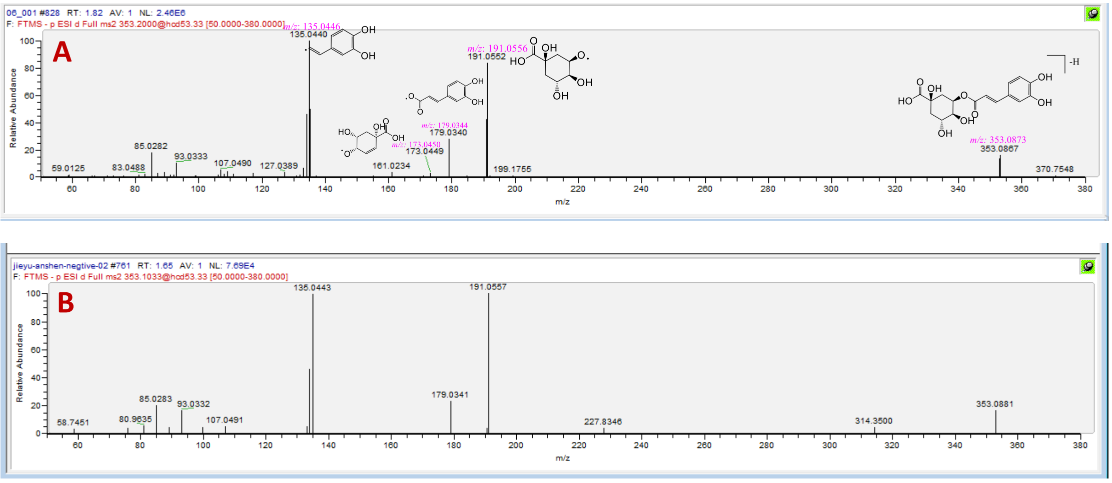

Fig. S7.1 The main results of standard 5-caffeoylquinicacid (CAS 906-33-2, $C_{16}H_{18}O_9$) and its corresponding peak in the TIC diagram using UPLC-Q-Orbitrap-MS analysis. (**A**) The MS/MS fragments of standard 5-caffeoylquinicacid. (**B**) The MS/MS spectra from chromatographic peak in R.T. 1.65 min in the *Jieyu Anshen* Granule extract.

Note: The *m/z* values in purple are the calculated ones. The *m/z* calculation was based on the relative atomic masses of C (12.0000), H (1.007825), O (15.994915), and N (14.003074)[1].

Identification: As seen in Fig. S7.1, the R.T. value, molecular ion peak, MS/MS spectra, and characteristic pears were highly similar. Thus, the chromatographic peak in R.T. 1.65 min in the *Jieyu Anshen* Granule extract was identified as 5-caffeoylquinicacid (CAS 906-33-2).

References:

[1] Gross., J.H., Mass spectrometry Beijing: Science press, 2013. 1.

Suppl. 8 Identification of caffeine (CAS 58-08-2, $C_8H_{10}N_4O_2$, M.W.,194.191).

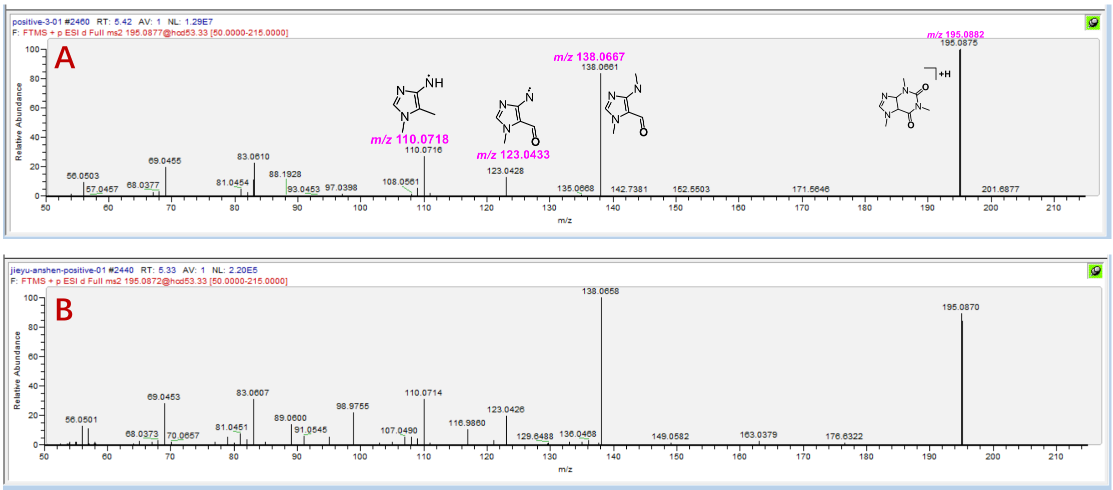

Fig. S8.1 The main results of standard caffeine (CAS 58-08-2, $C_8H_{10}N_4O_2$) and its corresponding peak in the TIC diagram using UPLC-Q-Orbitrap-MS analysis. (**A**) The MS/MS fragments of standard caffeine. (**B**) The MS/MS spectra from chromatographic peak in R.T. 5.33min in the *Jieyu Anshen* Granule extract.

Note: The *m/z* values in purple are the calculated ones. The *m/z* calculation was based on the relative atomic masses of C (12.0000), H (1.007825), O (15.994915), and N (14.003074)[1].

Identification: As seen in Fig. S8.1, the R.T. value, molecular ion peak, MS/MS spectra, and characteristic pears were highly similar. Thus, the chromatographic peak in R.T. 5.33 min in the *Jieyu Anshen* Granule extract was identified as caffeine (CAS 58-08-2, $C_8H_{10}N_4O_2$).

References:

[1] Gross., J.H., Mass spectrometry Beijing: Science press, 2013. 1.

Suppl. 9 Identification of trans-cinnamic acid (CAS 140-10-3, $C_9H_8O_2$, M.W., 148.159).

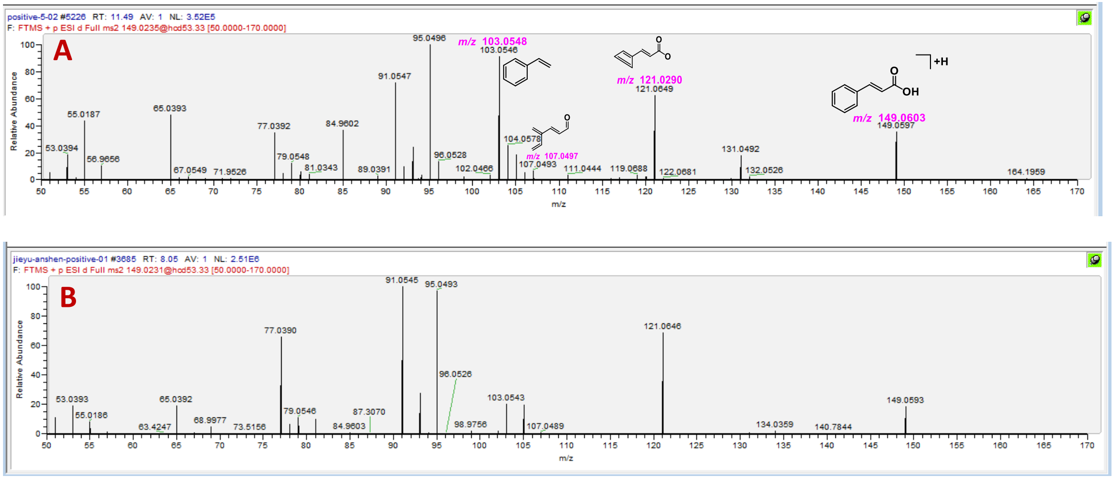

Fig. S9.1The main results of standard trans-cinnamic acid (CAS 140-10-3, $C_9H_8O_2$) and its corresponding peak in the TIC diagram using UPLC-Q-Orbitrap-MS analysis. (**A**) The MS/MS fragments of standard trans-cinnamic acid. (**B**) The MS/MS spectra from chromatographic peak in R.T. 8.05 min in the *Jieyu Anshen* Granule extract.

Note: The *m/z* values in purple are the calculated ones. The *m/z* calculation was based on the relative atomic masses of C (12.0000), H (1.007825), O (15.994915), and N (14.003074)[1].

Identification: As seen in Fig. S9.1 the R.T. value, molecular ion peak, MS/MS spectra, and characteristic pears were highly similar. Thus, the chromatographic peak in R.T. 8.05 min in the *Jieyu Anshen* Granule extract was identified as trans-cinnamic acid (CAS 140-10-3).

References:

[1] Gross., J.H., Mass spectrometry Beijing: Science press, 2013. 1.

Suppl. 10 Identification of geniposide (CAS 24512-63-8, $C_{17}H_{24}O_{10}$, M.W., 388.366)

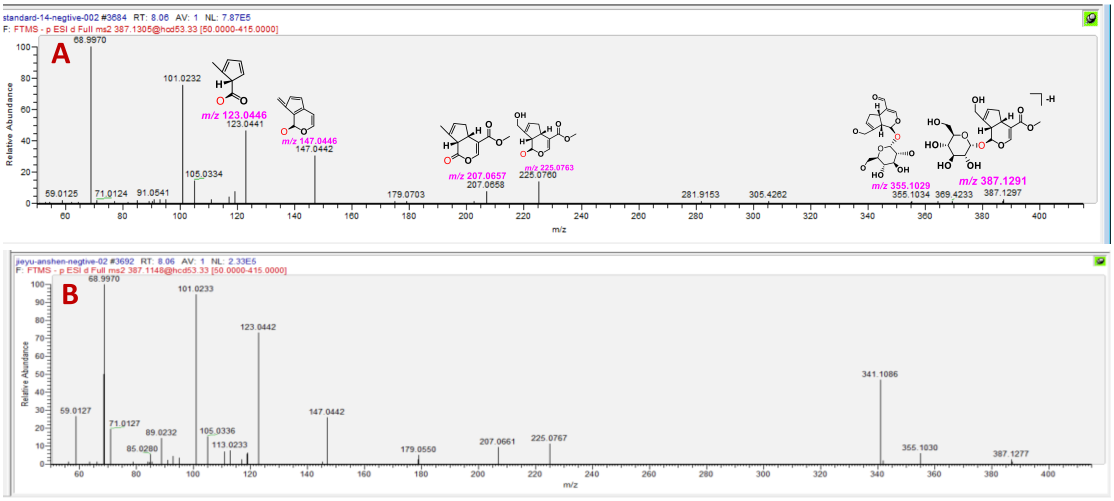

Fig. S10.1 The main results of standard geniposide (CAS 24512-63-8, $C_{17}H_{24}O_{10}$) and its corresponding peak in the TIC diagram using UPLC-Q-Orbitrap-MS analysis. (**A**) The MS/MS fragments of standard geniposide. (**B**) The MS/MS spectra from chromatographic peak in R.T. 8.06 min in the *Jieyu Anshen* Granule extract.

Note: The *m/z* values in purple are the calculated ones. The *m/z* calculation was based on the relative atomic masses of C (12.0000), H (1.007825), O (15.994915), and N (14.003074)[1].

Identification: As seen in Fig. S10.1, the R.T. value, molecular ion peak, MS/MS spectra, and characteristic pears were highly similar. In addition, Fig. S10.1 is highly similar like previous studies[2]. Thus, the chromatographic peak in R.T. 8.06 min in the *Jieyu Anshen* Granule extract was identified as geniposide (CAS 24512-63-8, $C_{17}H_{24}O_{10}$).

References:

1. Gross., J.H., Mass spectrometry Beijing: Science press, 2013. 1.
2. Li, X.B., et al., *Determination and pharmacokinetics of amygdalin in rats by LC-MS-MS.* J Chromatogr Sci, 2014. **52**(6): p. 476-81.

Suppl. 11 Identification of vicenin-2 (CAS 23666-13-9, $C_{27}H_{30}O_{15}$, M.W.,594.518).

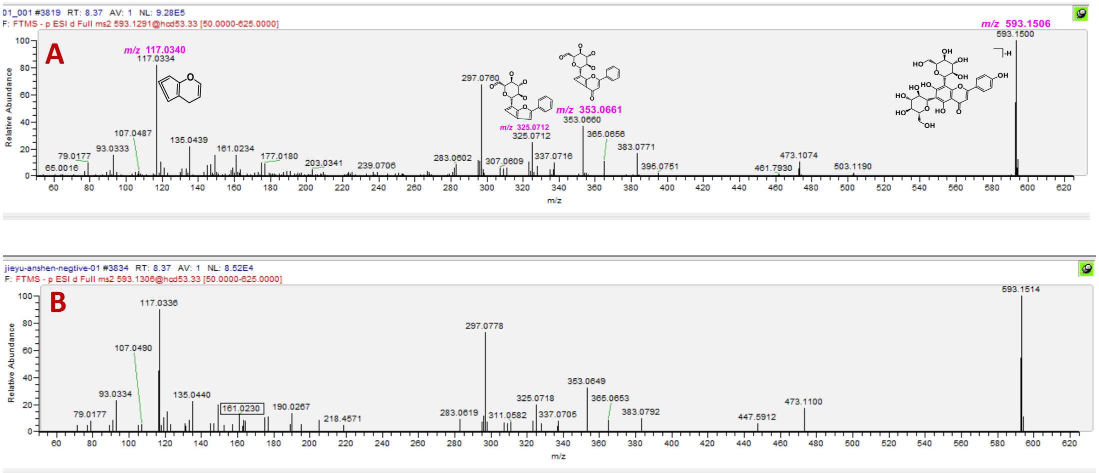

Fig. S11.1 The main results of standard vicenin-2 (CAS 23666-13-9, $C_{27}H_{30}O_{15}$) and its corresponding peak in the TIC diagram using UPLC-Q-Orbitrap-MS analysis. (**A**) The MS/MS fragments of standard vicenin-2. (**B**) The MS/MS spectra from chromatographic peak in R.T. 8.37 min in the *Jieyu* Anshen Granule extract.

Note: The *m/z* values in purple are the calculated ones. The *m/z* calculation was based on the relative atomic masses of C (12.0000), H (1.007825), O (15.994915), and N (14.003074)[1].

Identification: As seen in Fig. S11.1, the R.T. value, molecular ion peak, MS/MS spectra, and characteristic pears were highly similar. Thus, the chromatographic peak in R.T. 8.37 min in the *Jieyu Anshen* Granule extract was identified as vicenin-2 (CAS 23666-13-9).

References:

[1] Gross., J.H., Mass spectrometry Beijing: Science press, 2013. 1.

Suppl. 12 Identification of ferulic acid (CAS1135-24-6, $C_{10}H_{10}O_4$, M.W., 194.184).

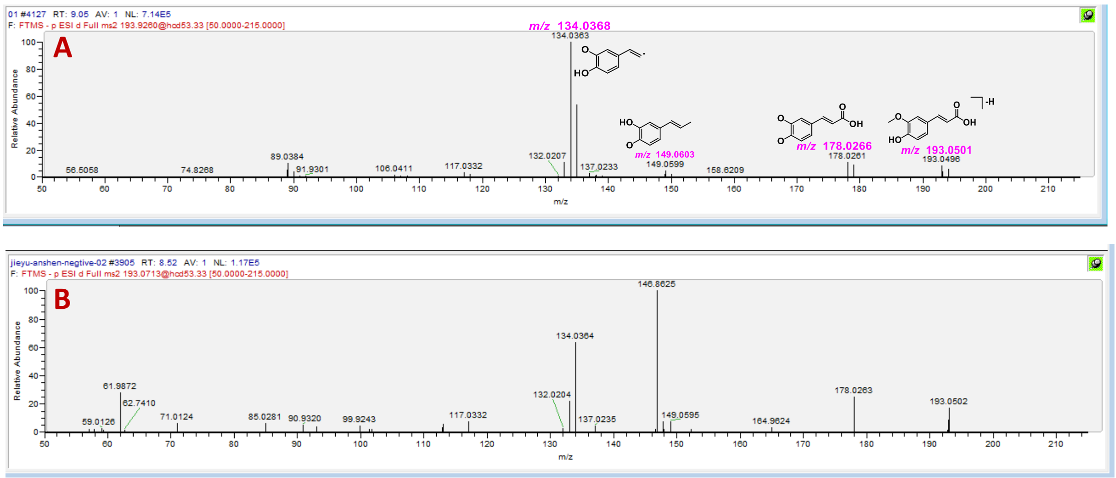

Fig. S12.1 The main results of standard ferulic acid (CAS1135-24-6, $C_{10}H_{10}O_4$) and its corresponding peak in the TIC diagram using UPLC-Q-Orbitrap-MS analysis. (**A**) The MS/MS fragments of standard ferulic acid. (**B**) The MS/MS spectra from chromatographic peak in R.T. 8.52 min in the *Jieyu Anshen* Granule extract.

Note: The *m/z* values in purple are the calculated ones. The *m/z* calculation was based on the relative atomic masses of C (12.0000), H (1.007825), O (15.994915), and N (14.003074)[1].

Identification: As seen in Fig. S12.1, the R.T. value, molecular ion peak, MS/MS spectra, and characteristic pears were highly similar. Thus, the chromatographic peak in R.T. 8.52 min in the *Jieyu Anshen* Granule extract was identified as ferulic acid (CAS1135-24-6, $C_{10}H_{10}O_4$).

References:

[1] Gross., J.H., Mass spectrometry Beijing: Science press, 2013. 1.

Suppl. 13 Identification of isoferulic acid (CAS 537-73-5, $C_{10}H_{10}O_4$, M.W., 194.184).

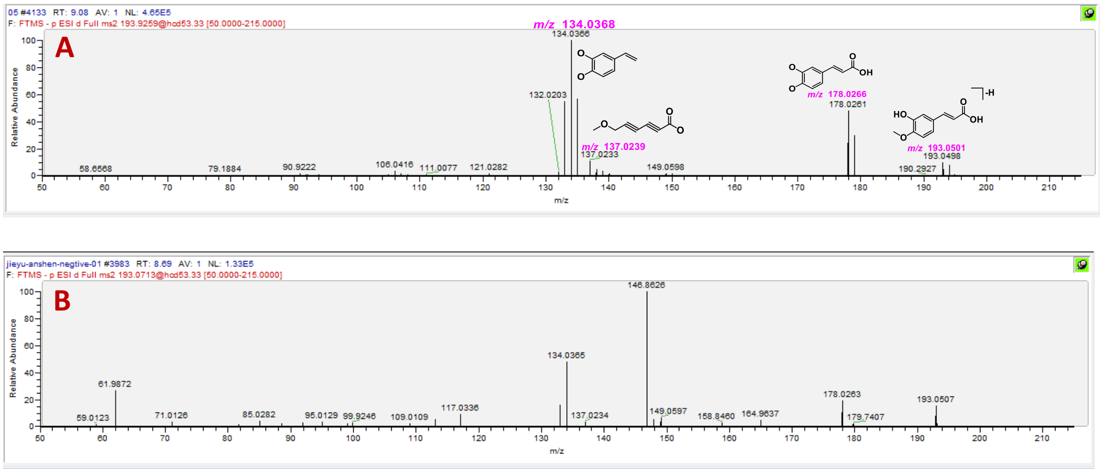

Fig. S13.1 The main results of standard isoferulic acid (CAS 537-73-5, $C_{10}H_{10}O_4$) and its corresponding peak in the TIC diagram using UPLC-Q-Orbitrap-MS analysis. (**A**) The MS/MS fragments of standard isoferulic acid. (**B**) The MS/MS spectra from chromatographic peak in R.T. 8.69 min in the *Jieyu Anshen* Granule extract.

Note: The *m/z* values in purple are the calculated ones. The *m/z* calculation was based on the relative atomic masses of C (12.0000), H (1.007825), O (15.994915), and N (14.003074)[1].

Identification: As seen in Fig. S13.1, the R.T. value, molecular ion peak, MS/MS spectra, and characteristic pears were highly similar. Thus, the chromatographic peak in R.T. 8.69 min in the *Jieyu Anshen* Granule extract was identified as isoferulic acid (CAS 537-73-5, $C_{10}H_{10}O_4$).

References:

[1] Gross., J.H., Mass spectrometry Beijing: Science press, 2013. 1.

Suppl. 14 Identification of schaftoside (CAS 51938-32-0, $C_{26}H_{28}O_{14}$, M.W., 564.49)

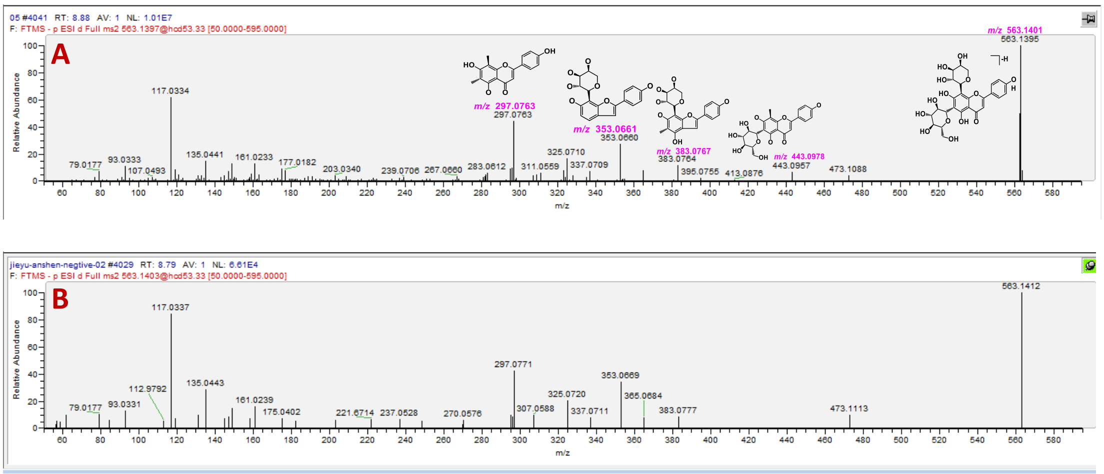

Fig. S14.1 The main results of standard schaftoside (CAS 51938-32-0, $C_{26}H_{28}O_{14}$) and its corresponding peak in the TIC diagram using UPLC-Q-Orbitrap-MS analysis. (**A**) The MS/MS fragments of standard schaftoside. (**B**) The MS/MS spectra from chromatographic peak in R.T. 8.79 min in the *Jieyu Anshen* Granule extract.

Note: The *m/z* values in purple are the calculated ones. The *m/z* calculation was based on the relative atomic masses of C (12.0000), H (1.007825), O (15.994915), and N (14.003074)[1].

Identification: As seen in Fig. S14.1, the R.T. value, molecular ion peak, MS/MS spectra, and characteristic pears were highly similar. Thus, the chromatographic peak in R.T. 8.79 min in the *Jieyu Anshen* Granule extract was identified as schaftoside (CAS 51938-32-0, $C_{26}H_{28}O_{14}$).

References:

[1] Gross., J.H., Mass spectrometry Beijing: Science press, 2013. 1.

Suppl. 15 Identification of lancerin (CAS 81991-99-3, $C_{19}H_{18}O_{10}$, M.W., 406.34).

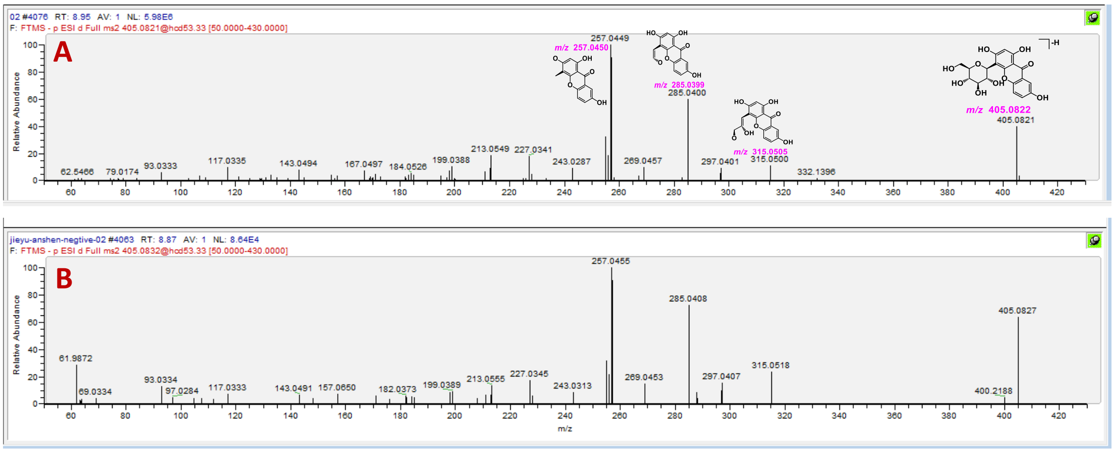

Fig. S15.1 The main results of standard lancerin (CAS 81991-99-3, $C_{19}H_{18}O_{10}$) and its corresponding peak in the TIC diagram using UPLC-Q-Orbitrap-MS analysis. (**A**) The MS/MS fragments of standard lancerin. (**B**) The MS/MS spectra from chromatographic peak in R.T. 8.87 min in the *Jieyu Anshen* Granule extract.

Note: The *m/z* values in purple are the calculated ones. The *m/z* calculation was based on the relative atomic masses of C (12.0000), H (1.007825), O (15.994915), and N (14.003074)[1].

Identification: As seen in Fig. S15.1, the R.T. value, molecular ion peak, MS/MS spectra, and characteristic pears were highly similar. Thus, the chromatographic peak in R.T. 8.87 min in the *Jieyu Anshen* Granule extract was identified as lancerin (CAS 81991-99-3, $C_{19}H_{18}O_{10}$).

References:

[1] Gross., J.H., Mass spectrometry Beijing: Science press, 2013. 1.

Suppl. 16 Identification of liquiritin (CAS 551-15-5, $C_{21}H_{22}O_9$)

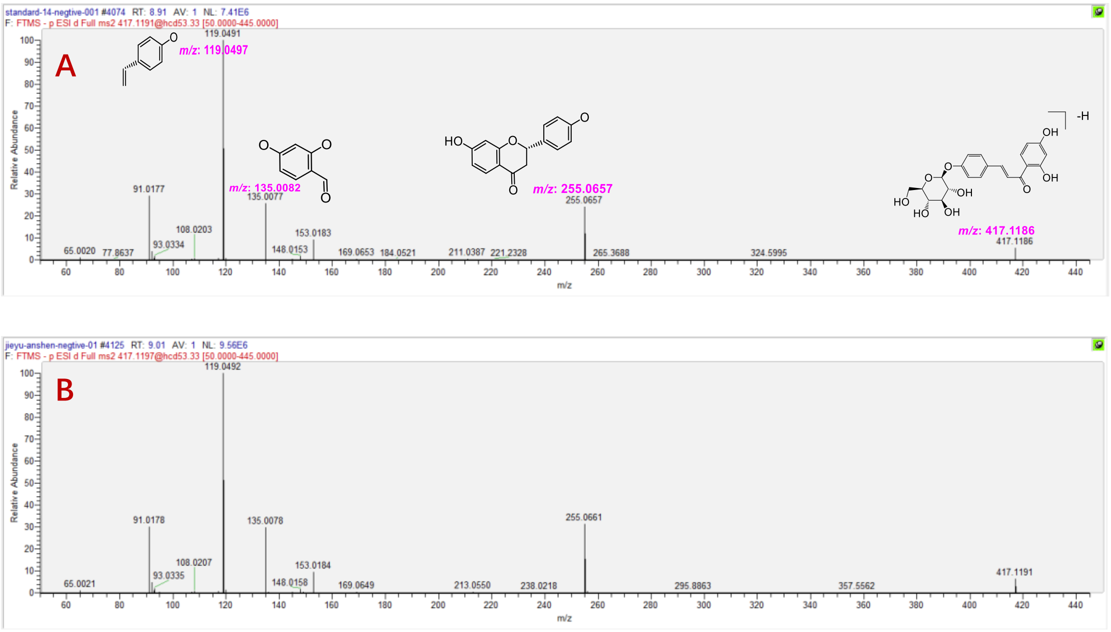

Fig. S16.1 The main results of standard liquiritin (CAS 551-15-5, $C_{21}H_{22}O_9$)and its corresponding peak in the TIC diagram using UPLC-Q-Orbitrap-MS analysis. (**A**) The MS/MS fragments of standard liquiritin. (**B**) The MS/MS spectra from chromatographic peak in R.T. 9.01 min in the *Jieyu Anshen* Granule extract.

Note: The *m/z* values in purple are the calculated ones. The *m/z* calculation was based on the relative atomic masses of C (12.0000), H (1.007825), O (15.994915), and N (14.003074)[1].

Identification: As seen in Fig. S16.1, the R.T. value, molecular ion peak, MS/MS spectra, and characteristic pears were highly similar. Thus, the chromatographic peak in R.T. 9.01 min in the *Jieyu Anshen* Granule extract was identified as liquiritin (CAS 551-15-5, $C_{21}H_{22}O_9$).

References

[1] Gross., J.H., Mass spectrometry Beijing: Science press, 2013. 1.

Suppl. 17 Identification of liquiritin apioside (CAS 74639-14-8, $C_{26}H_{30}O_{13}$)

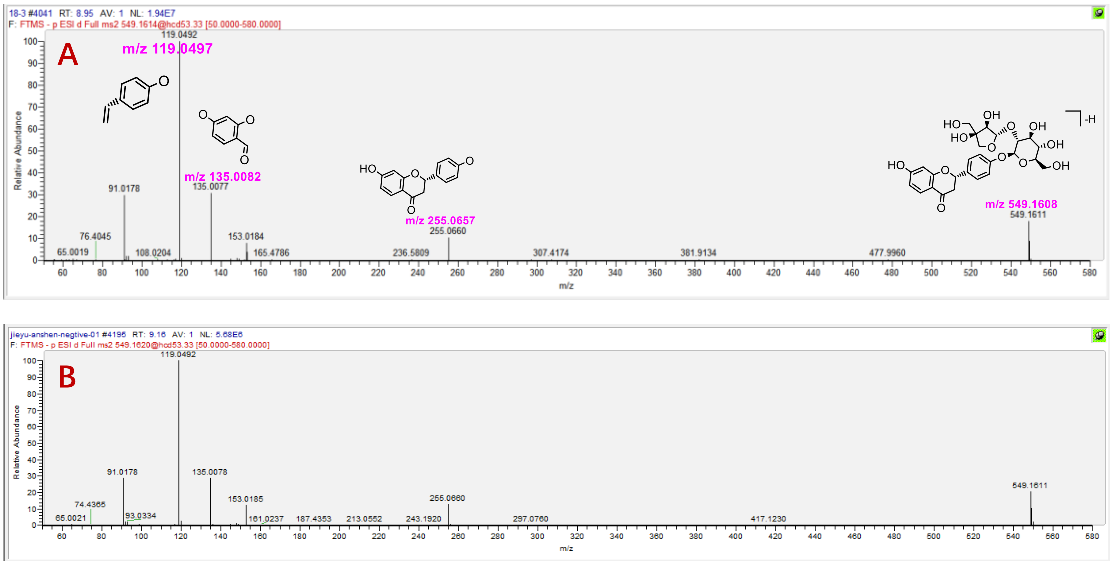

Fig. S17.1 The main results of standard liquiritin apioside (CAS 74639-14-8, $C_{26}H_{30}O_{13}$) and its corresponding peak in the TIC diagram using UPLC-Q-Orbitrap-MS analysis. (**A**) The MS/MS fragments of standard liquiritin apioside. (**B**) The MS/MS spectra from chromatographic peak in R.T. 9.16 min in the *Jieyu Anshen* Granule extract.

Note: The *m/z* values in purple are the calculated ones. The *m/z* calculation was based on the relative atomic masses of C (12.0000), H (1.007825), O (15.994915), and N (14.003074)[1].

Identification: As seen in Fig. S17.1, the R.T. value, molecular ion peak, MS/MS spectra, and characteristic pears were highly similar. Thus, the chromatographic peak in R.T. 9.16 min in the *Jieyu Anshen* Granule extract was identified as liquiritin apioside (CAS 74639-14-8, $C_{26}H_{30}O_{13}$).

References

[1] Gross., J.H., Mass spectrometry Beijing: Science press, 2013. 1.

Suppl. 18 Identification of 7-*O*–methylmangiferin (CAS 31002-12-7, $C_{20}H_{20}O_{11}$, M.W., 436.366).

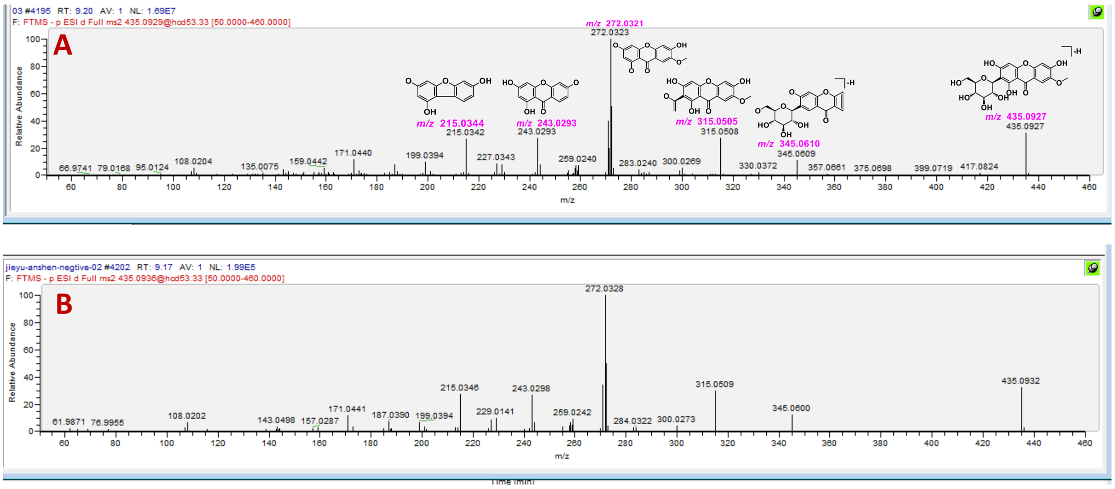

Fig. S18.1 The main results of standard 7-*O*-methylmangiferin (CAS 31002-12-7, $C_{20}H_{20}O_{11}$) and its corresponding peak in the TIC diagram using UPLC-Q-Orbitrap-MS analysis. (**A**) The MS/MS fragments of standard 7-*O*-methylmangiferin. (**B**) The MS/MS spectra from chromatographic peak in R.T. 9.17 min in the *Jieyu Anshen* Granule extract.

Note: The *m/z* values in purple are the calculated ones. The *m/z* calculation was based on the relative atomic masses of C (12.0000), H (1.007825), O (15.994915), and N (14.003074)[1].

Identification: As seen in Fig. S18.1, the R.T. value, molecular ion peak, MS/MS spectra, and characteristic pears were highly similar. Thus, the chromatographic peak in R.T. 9.17min in the *Jieyu Anshen* Granule extract was identified as 7-*O*-methylmangiferin (CAS 31002-12-7, $C_{20}H_{20}O_{11}$).

References:

[1] Gross., J.H., Mass spectrometry Beijing: Science press, 2013. 1.

Suppl. 19 Identification of Polygalaxanthone III (CAS 162857-78-5, $C_{25}H_{28}O_{15}$)

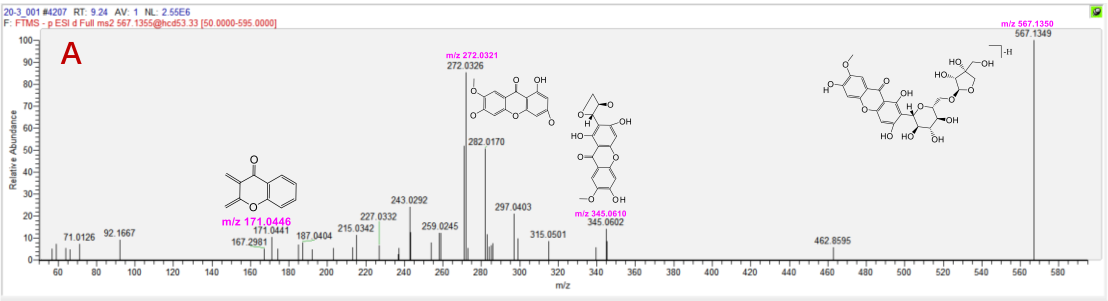

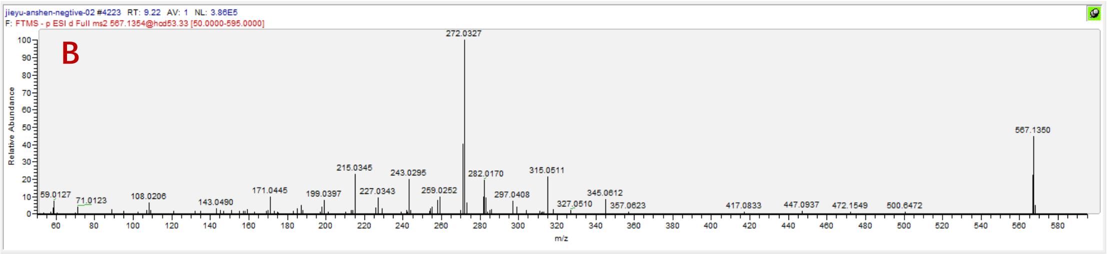

Fig. S19.1 The main results of standard polygalaxanthone III (CAS 162857-78-5, $C_{25}H_{28}O_{15}$) and its corresponding peak in the TIC diagram using UPLC-Q-Orbitrap-MS analysis. (**A**) The MS/MS fragments of standard polygalaxanthone III. (**B**) The MS/MS spectra from chromatographic peak in R.T. 9.22 min in the *Jieyu Anshen* Granule extract.

Note: The *m/z* values in purple are the calculated ones. The *m/z* calculation was based on the relative atomic masses of C (12.0000), H (1.007825), O (15.994915), and N (14.003074)[1].

Identification: As seen in Fig. S19.1, the R.T. value, molecular ion peak, MS/MS spectra, and characteristic pears were highly similar. Thus, the chromatographic peak in R.T. 9.22min in the *Jieyu Anshen* Granule extract was identified as Polygalaxanthone III (CAS 162857-78-5, $C_{25}H_{28}O_{15}$).

References:

[1] Gross., J.H., Mass spectrometry Beijing: Science press, 2013. 1.

Suppl. 20 Identification of swertisin (CAS 6991-10-2, $C_{22}H_{22}O_{10}$, M.W., 462.404).

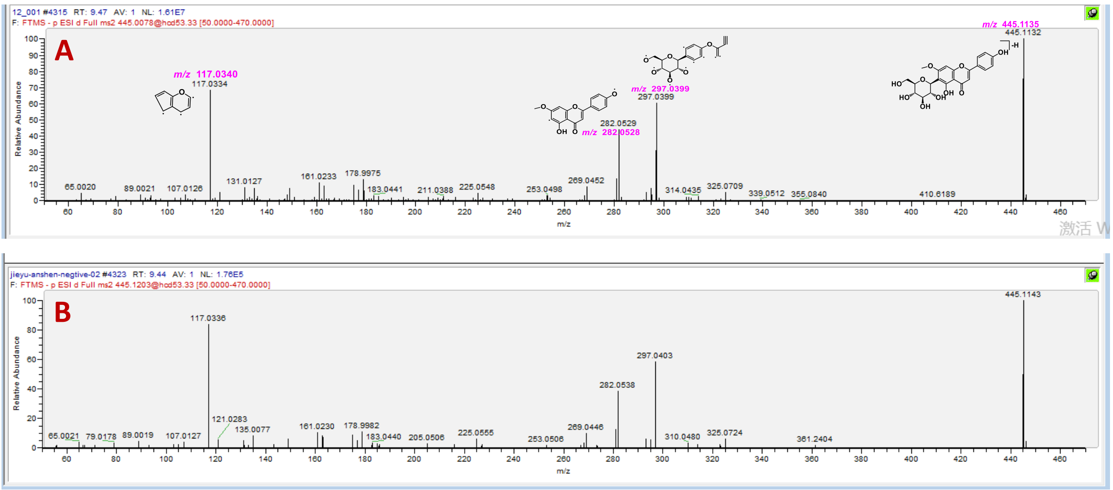

Fig. S20.1 The main results of standard swertisin (CAS 6991-10-2, $C_{22}H_{22}O_{10}$) and its corresponding peak in the TIC diagram using UPLC-Q-Orbitrap-MS analysis. (**A**) The MS/MS fragments of standard swertisin. (**B**) The MS/MS spectra from chromatographic peak in R.T. 9.44 min in the *Jieyu Anshen* Granule extract.

Note: The *m/z* values in purple are the calculated ones. The *m/z* calculation was based on the relative atomic masses of C (12.0000), H (1.007825), O (15.994915), and N (14.003074)[1].

Identification: As seen in Fig. S20.1, the R.T. value, molecular ion peak, MS/MS spectra, and characteristic pears were highly similar. Thus, the chromatographic peak in R.T 9.44 min in the *Jieyu Anshen* Granule extract was identified as swertisin (CAS 6991-10-2, $C_{22}H_{22}O_{10}$).

References

[1] Gross., J.H., Mass spectrometry Beijing: Science press, 2013. 1.

Suppl. 21 Identification of isoviolanthin (CAS 40788-84-9, $C_{27}H_{30}O_{14}$, M.W., 578.519).

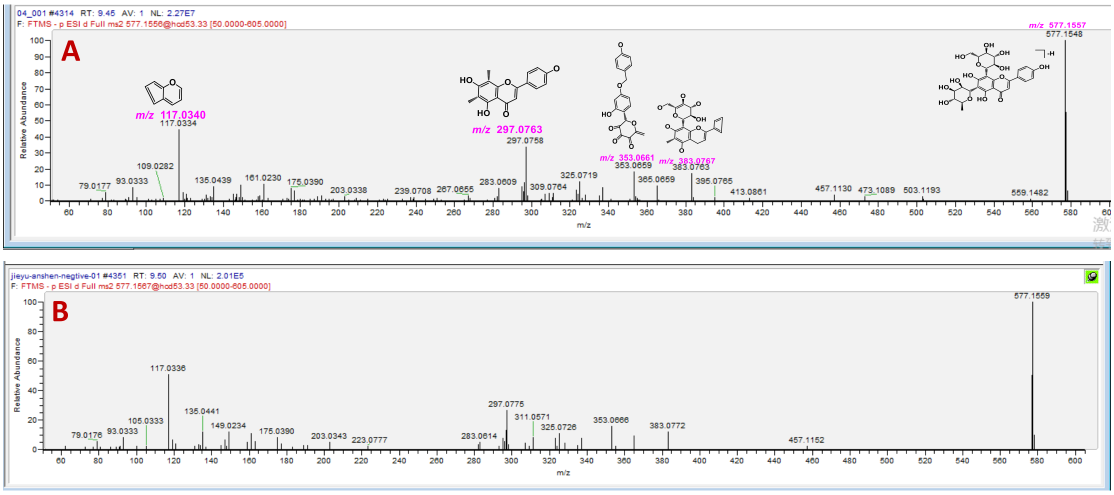

Fig. S21.1 The main results of standard isoviolanthin (CAS 40788-84-9, $C_{27}H_{30}O_{14}$) and its corresponding peak in the TIC diagram using UPLC-Q-Orbitrap-MS analysis. (**A**) The MS/MS fragments of standard isoviolanthin. (**B**) The MS/MS spectra from chromatographic peak in R.T. 9.50 min in the *Jieyu Anshen* Granule extract.

Note: The *m/z* values in purple are the calculated ones. The *m/z* calculation was based on the relative atomic masses of C (12.0000), H (1.007825), O (15.994915), and N (14.003074)[1].

Identification: As seen in Fig. S21.1, the R.T. value, molecular ion peak, MS/MS spectra, and characteristic pears were highly similar. Thus, the chromatographic peak in R.T. 9.50 min in the *Jieyu Anshen* Granule extract was identified as isoviolanthin (CAS 40788-84-9, $C_{27}H_{30}O_{14}$).

References

[1] Gross., J.H., Mass spectrometry Beijing: Science press, 2013. 1.

Suppl. 22 Identification of rutin (CAS 153-18-4, $C_{27}H_{30}O_{16}$, M.W. 610.518).

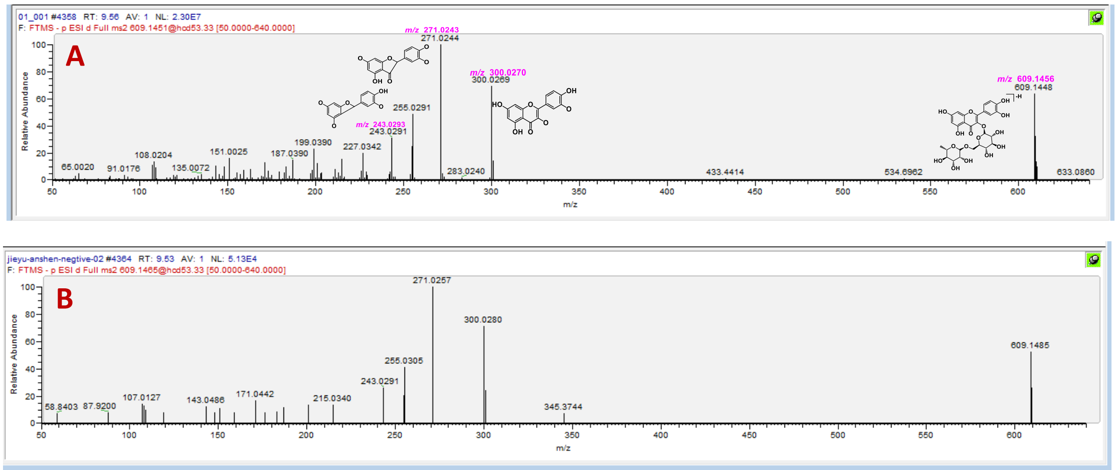

Fig. S22.2 The main results of standard rutin (CAS 153-18-4, $C_{27}H_{30}O_{16}$) and its corresponding peak in the TIC diagram using UPLC-Q-Orbitrap-MS analysis. (**A**) The MS/MS fragments of standard rutin. (**B**) The MS/MS spectra from chromatographic peak in R.T. 9.53 min in the *Jieyu Anshen* Granule extract.

Note：The *m/z* values in purple are the calculated ones. The *m/z* calculation was based on the relative atomic masses of C (12.0000), H (1.007825), O (15.994915), and N (14.003074)[1].

Identification: As seen in Fig. S22.2, the R.T. value, molecular ion peak, MS/MS spectra, and characteristic pears were highly similar. Thus, the chromatographic peak in R.T. 9.53 min in the *Jieyu Anshen* Granule extract was identified as rutin (CAS 153-18-4, $C_{27}H_{30}O_{16}$).

References

[1] Gross., J.H., Mass spectrometry Beijing: Science press, 2013. 1.

Suppl. 23 Identification of naringenin-7-*O*-β-D-glucoside (CAS 529-55-5, $C_{21}H_{22}O_{10}$ M.W., 434.393).

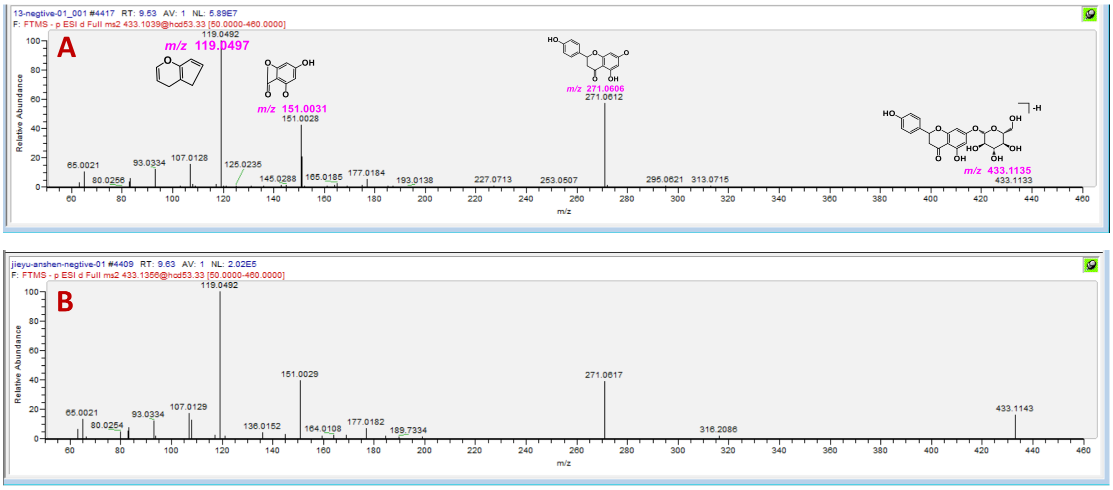

Fig. S23.1 The main results of standard naringenin-7-*O*-β-D-glucoside (CAS 529-55-5, $C_{21}H_{22}O_{10}$) and its corresponding peak in the TIC diagram using UPLC-Q-Orbitrap-MS analysis. (**A**) The MS/MS fragments of standard naringenin-7-*O*-β-D-glucoside. (**B**) The MS/MS spectra from chromatographic peak in R.T. 9.63 min in the *Jieyu Anshen* Granule extract.

Note: The *m/z* values in purple are the calculated ones. The *m/z* calculation was based on the relative atomic masses of C (12.0000), H (1.007825), O (15.994915), and N (14.003074)[1].

Identification: As seen in Fig. S23.1, the R.T. value, molecular ion peak, MS/MS spectra, and characteristic pears were highly similar. Thus, the chromatographic peak in R.T. 9.63 min in the *Jieyu Anshen* Granule extract was identified as naringenin-7-*O*-β-D-glucoside (CAS 529-55-5, $C_{21}H_{22}O_{10}$).

References

[1] Gross., J.H., Mass spectrometry Beijing: Science press, 2013. 1.

Suppl. 24 Identification of Isoliquiritin (CAS 5041-81-6, $C_{21}H_{22}O_9$)

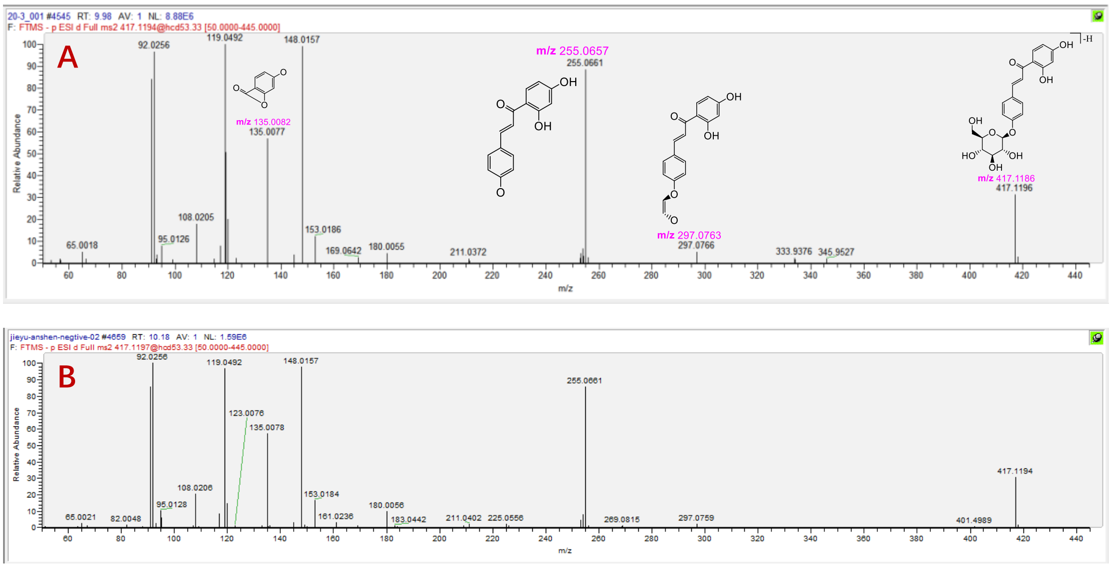

Fig. S24.1 The main results of standard isoliquiritin (CAS 5041-81-6, $C_{21}H_{22}O_9$) and its corresponding peak in the TIC diagram using UPLC-Q-Orbitrap-MS analysis. (**A**) The MS/MS fragments of standard isoliquiritin. (**B**) The MS/MS spectra from chromatographic peak in R.T. 10.18 min in the *Jieyu Anshen* Granule extract.

Note: The *m/z* values in purple are the calculated ones. The *m/z* calculation was based on the relative atomic masses of C (12.0000), H (1.007825), O (15.994915), and N (14.003074)[1].

Identification: As seen in Fig. S24.1, the R.T. value, molecular ion peak, MS/MS spectra, and characteristic pears were highly similar. Thus, the chromatographic peak in R.T. 10.18 min in the *Jieyu Anshen* Granule extract was identified as isoliquiritin (CAS 5041-81-6, $C_{21}H_{22}O_9$).

References

[1] Gross., J.H., Mass spectrometry Beijing: Science press, 2013. 1.

Suppl. 25 Identification of liquiritigenin (CAS 578-86-9, $C_{15}H_{12}O_4$)

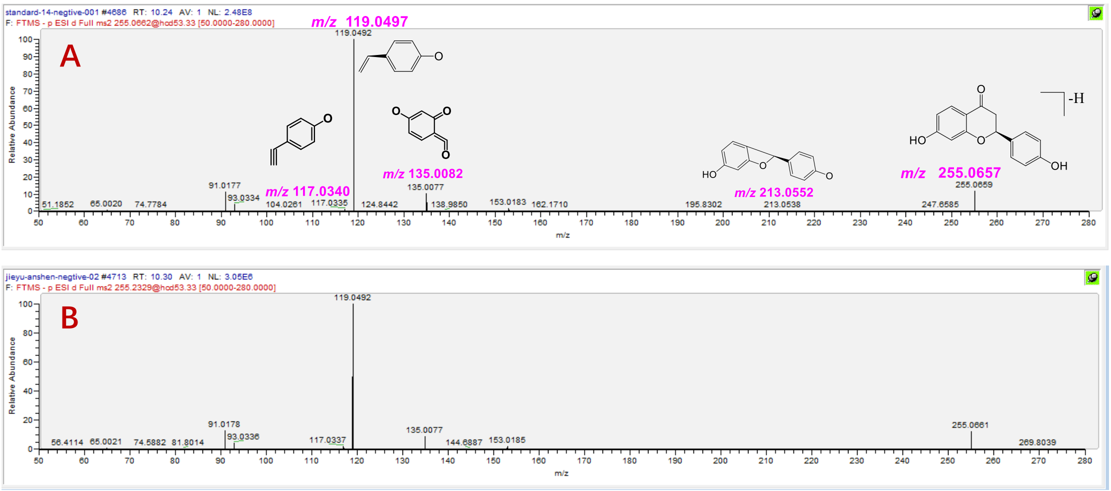

Fig. S25.1 The main results of standard liquiritigenin (CAS 578-86-9, $C_{15}H_{12}O_4$) and its corresponding peak in the TIC diagram using UPLC-Q-Orbitrap-MS analysis. (**A**) The MS/MS fragments of standard isoliquiritin. (**B**) The MS/MS spectra from chromatographic peak in R.T. 10.30 min in the *Jieyu Anshen* Granule extract.

Note: The *m/z* values in purple are the calculated ones. The *m/z* calculation was based on the relative atomic masses of C (12.0000), H (1.007825), O (15.994915), and N (14.003074)[1].

Identification: As seen in Fig. S25.1, the R.T. value, molecular ion peak, MS/MS spectra, and characteristic pears were highly similar. Thus, the chromatographic peak in R.T. 10.30 min in the *Jieyu Anshen* Granule extract was identified as liquiritigenin (CAS 578-86-9, $C_{15}H_{12}O_4$).

References

[1] Gross., J.H., Mass spectrometry Beijing: Science press, 2013. 1.

Suppl. 26 Identification of chrysoeriol (CAS 491-71-4, $C_{16}H_{12}O_6$, M.W., 300.263).

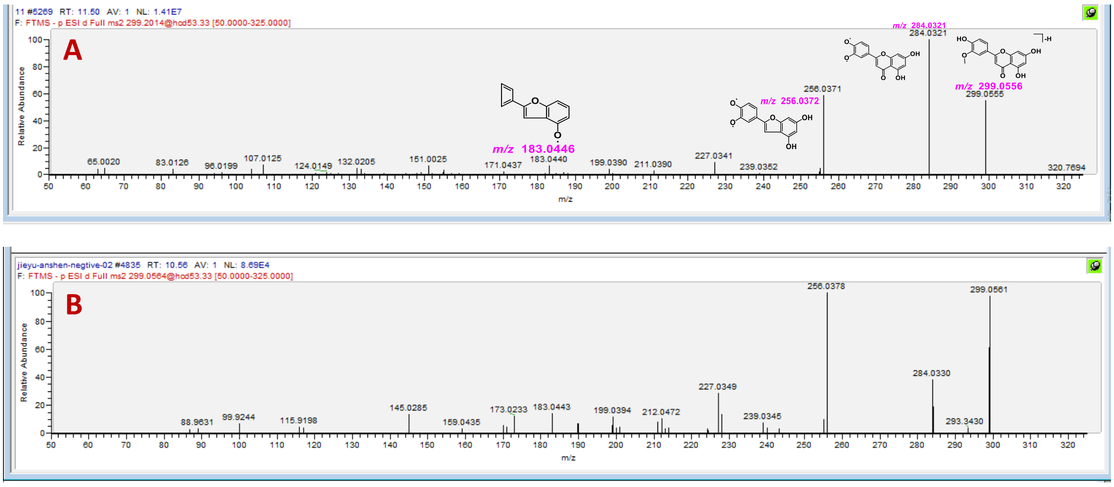

Fig. S26.1 The main results of standard chrysoeriol (CAS 491-71-4, $C_{16}H_{12}O_6$) and its corresponding peak in the TIC diagram using UPLC-Q-Orbitrap-MS analysis. (**A**) The MS/MS fragments of standard chrysoeriol. (**B**) The MS/MS spectra from chromatographic peak in R.T. 10.56 min in the *Jieyu Anshen* Granule extract.

Note: The *m/z* values in purple are the calculated ones. The *m/z* calculation was based on the relative atomic masses of C (12.0000), H (1.007825), O (15.994915), and N (14.003074)[1].

Identification: As seen in Fig. S26.1, the R.T. value, molecular ion peak, MS/MS spectra, and characteristic pears were highly similar. Thus, the chromatographic peak in R.T. 10.56 min in the *Jieyu Anshen* Granule extract was identified as chrysoeriol (CAS 491-71-4, $C_{16}H_{12}O_6$).

References:

[1] Gross., J.H., Mass spectrometry Beijing: Science press, 2013. 1.

Suppl. 27 Identification of 7,4'-dihydroxyflavone (CAS 2196-14-7, $C_{15}H_{10}O_4$, M.W., 254.238).

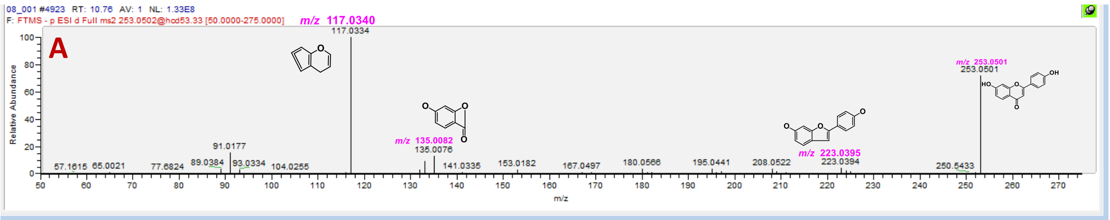

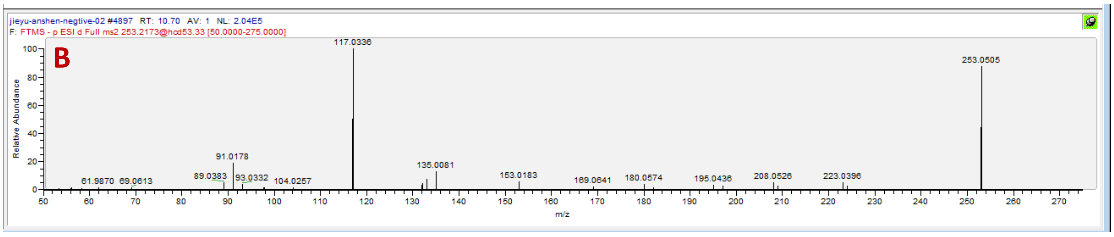

Fig. S27.1 The main results of standard7,4'-dihydroxyflavone (CAS 2196-14-7, $C_{15}H_{10}O_4$) and its corresponding peak in the TIC diagram using UPLC-Q-Orbitrap-MS analysis. (**A**) The MS/MS fragments of standard 7,4'-dihydroxyflavone. (**B**) The MS/MS spectra from chromatographic peak in R.T. 10.70 min in the *Jieyu Anshen* Granule extract.

Note: The *m/z* values in purple are the calculated ones. The *m/z* calculation was based on the relative atomic masses of C (12.0000), H (1.007825), O (15.994915), and N (14.003074)[1].

Identification: As seen in Fig. S27.1, the R.T. value, molecular ion peak, MS/MS spectra, and characteristic pears were highly similar. Thus, the chromatographic peak in R.T. 10.70 min in the *Jieyu Anshen* Granule extract was identified as 7,4'-dihydroxyflavone (CAS 2196-14-7, $C_{15}H_{10}O_4$).

References:

[1] Gross., J.H., Mass spectrometry Beijing: Science press, 2013. 1.

Suppl. 28 Identification of calycosin (CAS 20575-57-9, $C_{16}H_{12}O_5$, M.W.,284.263).

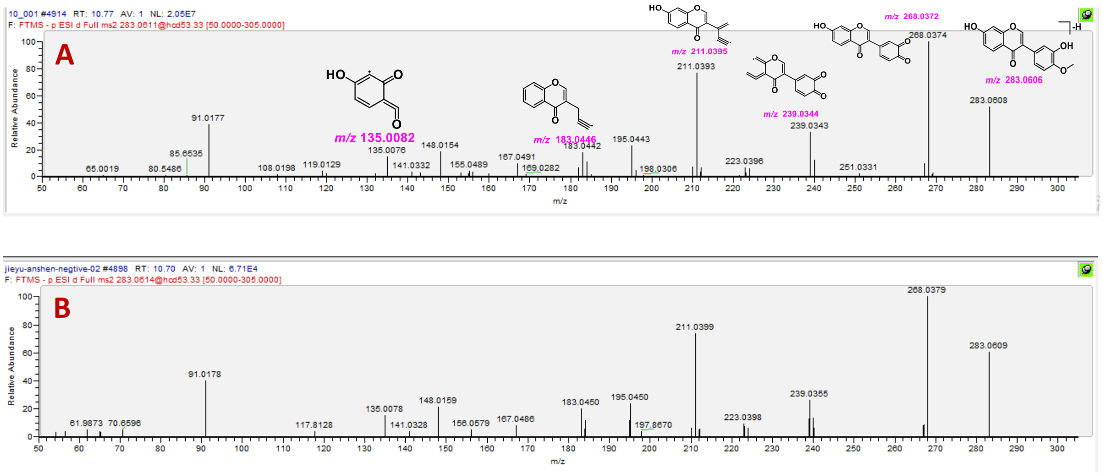

Fig. S28.1The main results of standard calycosin (CAS 20575-57-9, $C_{16}H_{12}O_5$) and its corresponding peak in the TIC diagram using UPLC-Q-Orbitrap-MS analysis. (**A**) The typical chromatographic peak (upper) and MS/MS fragments (below) of standard calycosin. (**B**) The MS/MS spectra from chromatographic peak in the *Jieyu Anshen* Granule extract.

Note: The *m/z* values in purple are the calculated ones. The *m/z* calculation was based on the relative atomic masses of C (12.0000), H (1.007825), O (15.994915), and N (14.003074)[1].

Identification: As seen in Fig. S28.1 the R.T. value, molecular ion peak, MS/MS spectra, and characteristic pears were highly similar. Thus, the chromatographic peak in the *Jieyu Anshen* Granule extract was identified as calycosin (CAS 20575-57-9, $C_{16}H_{12}O_5$).

References:

[1] Gross., J.H., Mass spectrometry Beijing: Science press, 2013. 1.

Suppl. 29 Identification of naringenin (CAS 480-41-1, $C_{15}H_{12}O_5$, M.W. 272.253).

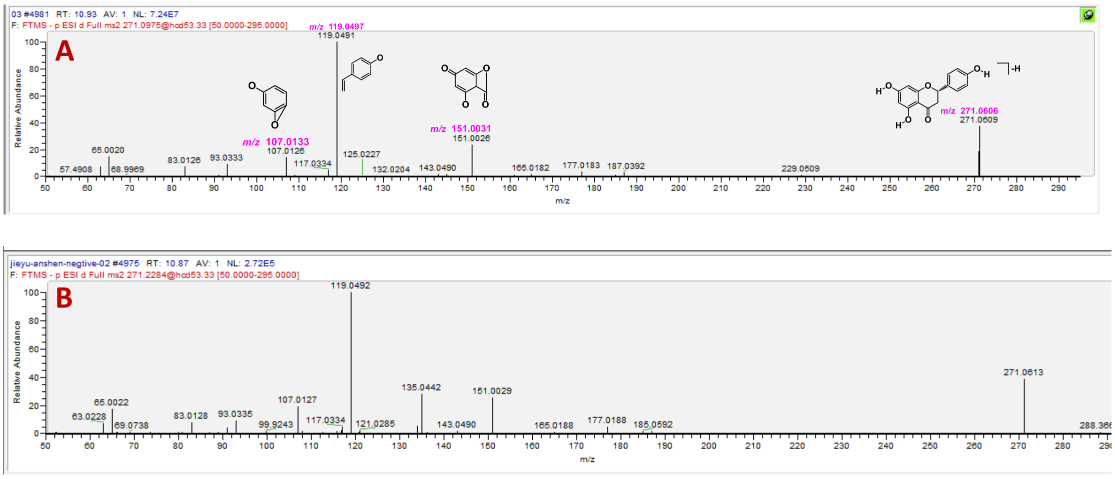

Fig. S29.1The main results of standard naringenin (CAS 480-41-1, $C_{15}H_{12}O_5$) and its corresponding peak in the TIC diagram using UPLC-Q-Orbitrap-MS analysis. (**A**) The MS/MS fragments of standard naringenin. (**B**) The MS/MS spectra from chromatographic peak in R.T. 10.87 min in the *Jieyu Anshen* Granule extract.

Note: The *m/z* values in purple are the calculated ones. The *m/z* calculation was based on the relative atomic masses of C (12.0000), H (1.007825), O (15.994915), and N (14.003074)[1].

Identification: As seen in Fig. S29.1 the R.T. value, molecular ion peak, MS/MS spectra, and characteristic pears were highly similar. Thus, the chromatographic peak in R.T. 10.87 min in the *Jieyu Anshen* Granule extract was identified as naringenin (CAS 480-41-1, $C_{15}H_{12}O_5$).

References:

[1] Gross., J.H., Mass spectrometry Beijing: Science press, 2013. 1.

Suppl. 30 Identification of isoliquiritigenin (CAS 961-29-5, $C_{15}H_{12}O_4$, M.W. 256.253).

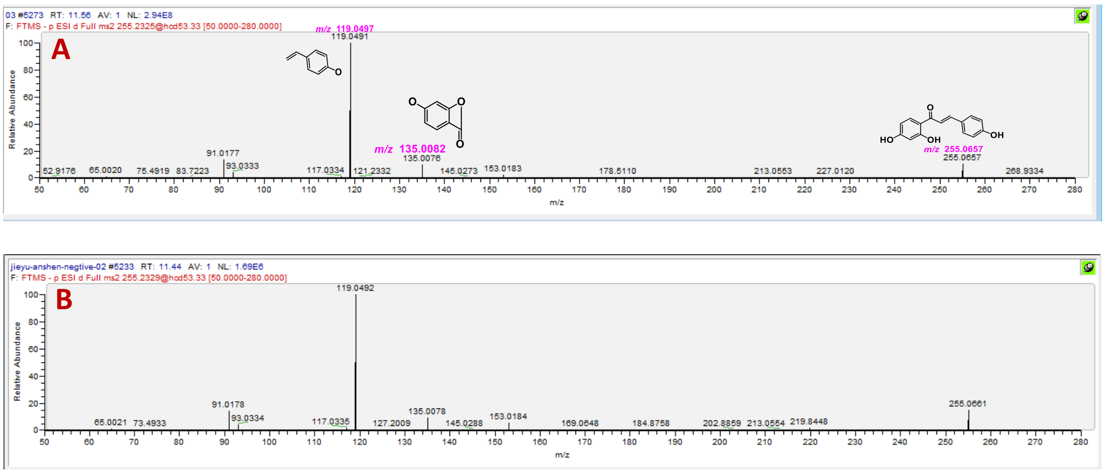

Fig. S30.1 The main results of standard isoliquiritigenin (CAS 961-29-5, $C_{15}H_{12}O_4$) and its corresponding peak in the TIC diagram using UPLC-Q-Orbitrap-MS analysis. (**A**) The MS/MS fragments of standard isoliquiritigenin. (**B**) The MS/MS spectra from chromatographic peak in R.T. 11.44 min in the *Jieyu Anshen* Granule extract.

Note: The *m/z* values in purple are the calculated ones. The *m/z* calculation was based on the relative atomic masses of C (12.0000), H (1.007825), O (15.994915), and N (14.003074)[1].

Identification: As seen in Fig. S30.1, the R.T. value, molecular ion peak, MS/MS spectra, and characteristic pears were highly similar. Thus, the chromatographic peak in R.T. 11.44 min in the *Jieyu Anshen* Granule extract was identified as isoliquiritigenin (CAS 961-29-5, $C_{15}H_{12}O_4$).

References:

[1] Gross., J.H., Mass spectrometry Beijing: Science press, 2013. 1.

Suppl. 31 Identification of calycosin-7-O-β-D-glucoside (CAS 20633-67-4, M.W. $C_{22}H_{22}O_{10}$)

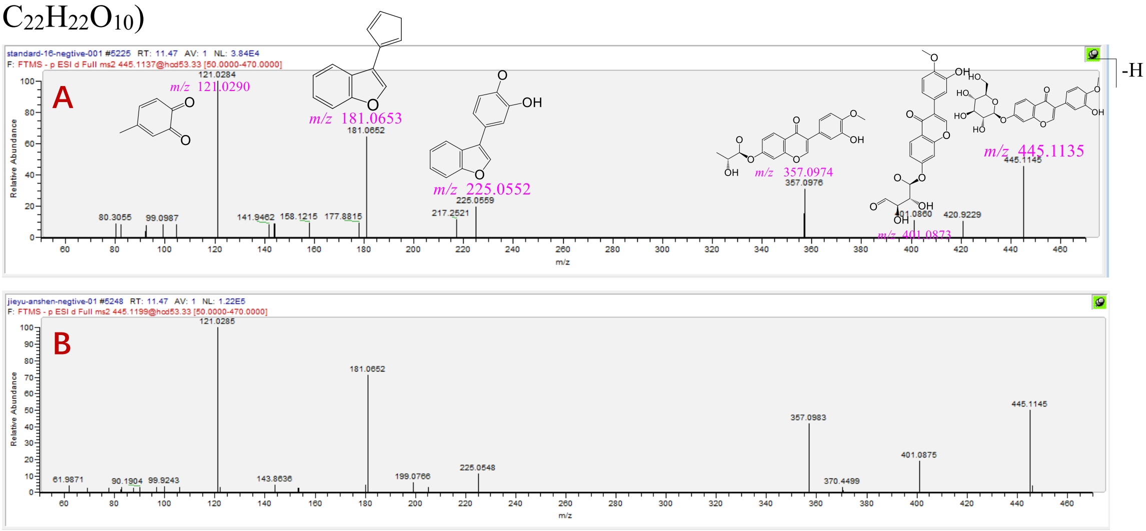

Fig. S31.1 The main results of standard calycosin-7-*O*-β-D-glucoside (CAS 23180-57-6, $C_{22}H_{22}O_{10}$) and its corresponding peak in the TIC diagram using UPLC-Q-Orbitrap-MS analysis. (**A**) The MS/MS fragments of standard calycosin-7-O-β-D-glucoside. (**B**) The MS/MS spectra from chromatographic peak in R.T. 11.47 min in the *Jieyu Anshen* Granule extract.

Note: The *m/z* values in purple are the calculated ones. The *m/z* calculation was based on the relative atomic masses of C (12.0000), H (1.007825), O (15.994915), and N (14.003074)[1].

Identification: As seen in Fig. S31.1, the R.T. value, molecular ion peak, MS/MS spectra, and characteristic pears were highly similar. Thus, the chromatographic peak in R.T. 11.44 min in the *Jieyu Anshen* Granule extract was identified as calycosin-7-O-β-D-glucoside (CAS 23180-57-6, $C_{22}H_{22}O_{10}$).

References:

[1] Gross., J.H., Mass spectrometry Beijing: Science press, 2013. 1.

Suppl. 32 Identification of formononetin (CAS 485-72-3, $C_{16}H_{12}O_4$, M.W., 268.264).

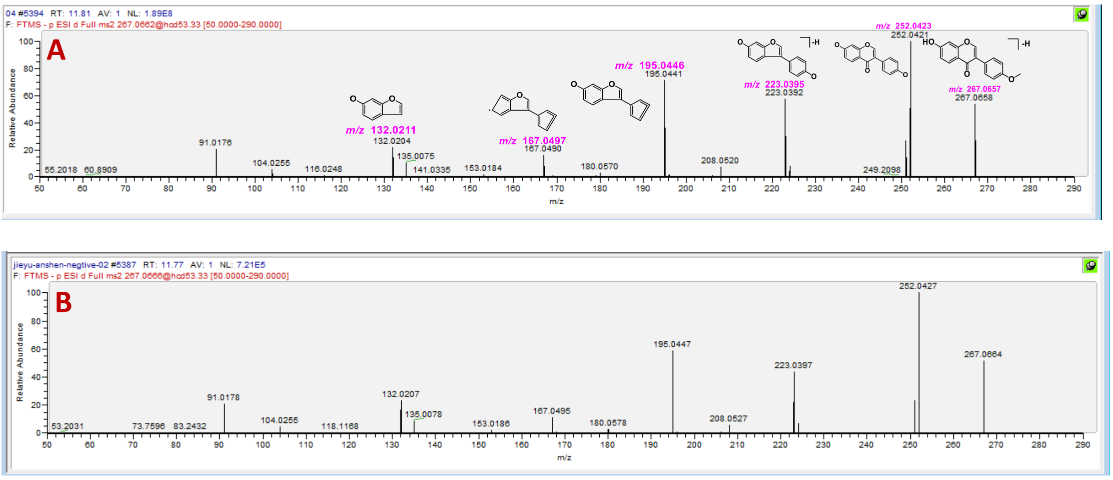

Fig. S32.1 The main results of standard formononetin (CAS 485-72-3, $C_{16}H_{12}O_4$) and its corresponding peak in the TIC diagram using UPLC-Q-Orbitrap-MS analysis. (**A**) The MS/MS fragments of standard formononetin. (**B**) The MS/MS spectra from chromatographic peak in R.T. 11.77 min in the *Jieyu Anshen* Granule extract.

Note: The *m/z* values in purple are the calculated ones. The *m/z* calculation was based on the relative atomic masses of C (12.0000), H (1.007825), O (15.994915), and N (14.003074)[1].

Identification: As seen in Fig. S32.1, the R.T. value, molecular ion peak, MS/MS spectra, and characteristic pears were highly similar. Thus, the chromatographic peak in R.T. 11.77 min in the *Jieyu Anshen* Granule extract was identified as formononetin (CAS 485-72-3, $C_{16}H_{12}O_4$).

References:

[1] Gross., J.H., Mass spectrometry Beijing: Science press, 2013. 1.

Suppl. 33 Identification of 1,2,3,7-tetramethoxyxanthone (CAS 22804-52-0, $C_{17}H_{16}O_6$)

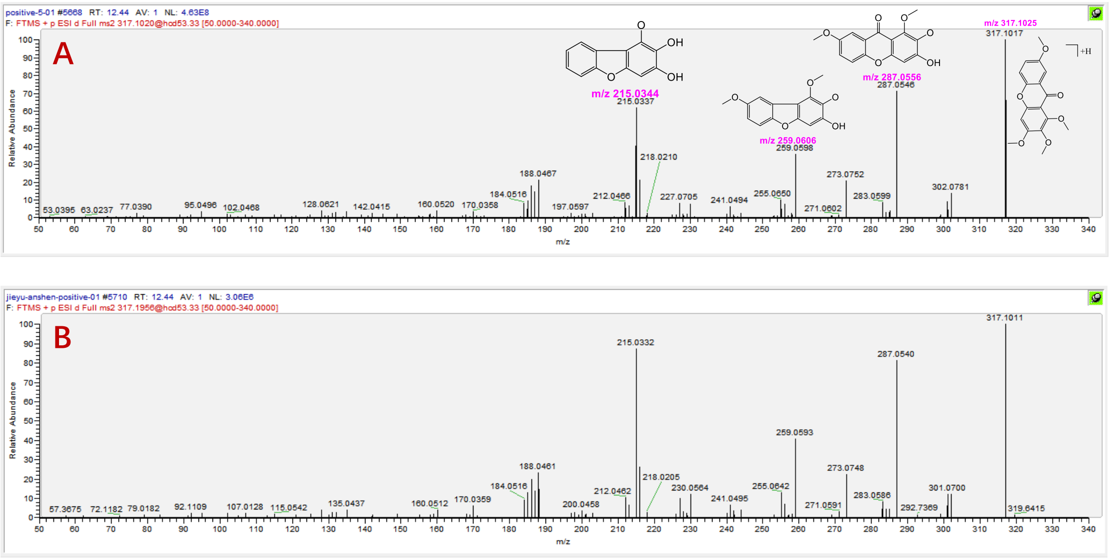

Fig. S33.1 The main results of standard 1,2,3,7-tetramethoxyxanthone (CAS 22804-52-0, $C_{17}H_{16}O_6$) and its corresponding peak in the TIC diagram using UPLC-Q-Orbitrap-MS analysis. (**A**) The MS/MS fragments of standard 1,2,3,7-tetramethoxyxanthone. (**B**) The MS/MS spectra from chromatographic peak in R.T. 12.44 min in the *Jieyu Anshen* Granule extract.

Note: The *m/z* values in purple are the calculated ones. The *m/z* calculation was based on the relative atomic masses of C (12.0000), H (1.007825), O (15.994915), and N (14.003074)[1].

Identification: As seen in Fig. S33.1, the R.T. value, molecular ion peak, MS/MS spectra, and characteristic pears were highly similar. Thus, the chromatographic peak in R.T. 12.44 min in the *Jieyu Anshen* Granule extract was identified as 1,2,3,7-tetramethoxyxanthone (CAS 22804-52-0, $C_{17}H_{16}O_6$).

Reference

[1] Gross., J.H., Mass spectrometry Beijing: Science press, 2013. 1.

Suppl. 34 Identification of 3,5,6,7,8,3’,4’-heptemthoxyflavone (CAS1178-24-1, $C_{22}H_{24}O_9$, M.W.432.421).

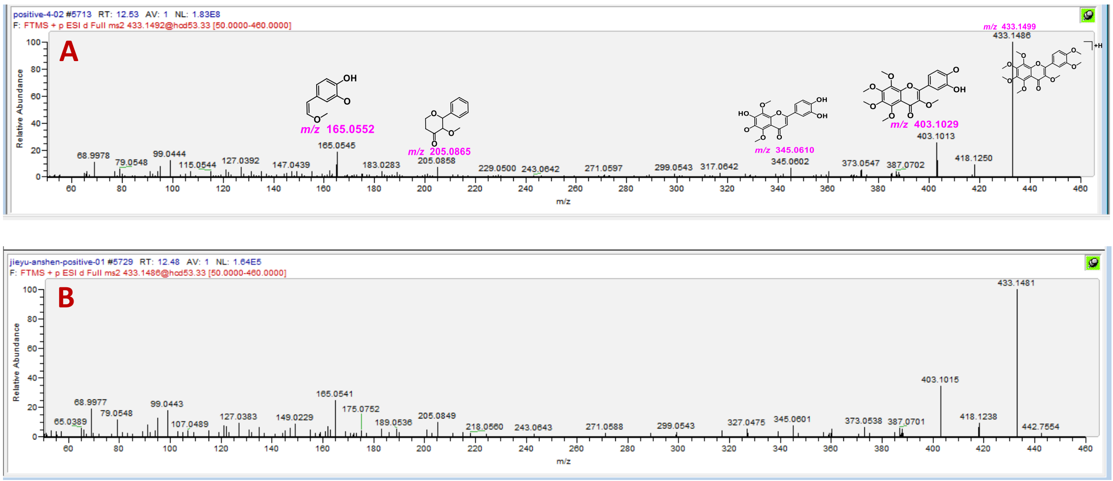

Fig. S34.1 The main results of standard 3,5,6,7,8,3’,4’- heptemthoxyflavone (CAS1178-24-1, $C_{22}H_{24}O_9$) and its corresponding peak in the TIC diagram using UPLC-Q-Orbitrap-MS analysis. (**A**) The MS/MS fragments of standard 3,5,6,7,8,3’,4’-heptemthoxyflavone. (**B**) The MS/MS spectra from chromatographic peak in R.T. 12.48 min in the *Jieyu Anshen* Granule extract.

Note: The *m/z* values in purple are the calculated ones. The *m/z* calculation was based on the relative atomic masses of C (12.0000), H (1.007825), O (15.994915), and N (14.003074)[1].

Identification: As seen in Fig. S34.1, the R.T. value, molecular ion peak, MS/MS spectra, and characteristic pears were highly similar. Thus, the chromatographic peak in R.T. 12.48min in the *Jieyu Anshen* Granule extract was identified as 3,5,6,7,8,3’,4’-heptemthoxyflavone (CAS1178-24-1, $C_{22}H_{24}O_9$).

References:

[1] Gross., J.H., Mass spectrometry Beijing: Science press, 2013. 1.

Suppl. 35 Identification of ligustilide (CAS 81944-09-4, $C_{12}H_{14}O_2$)

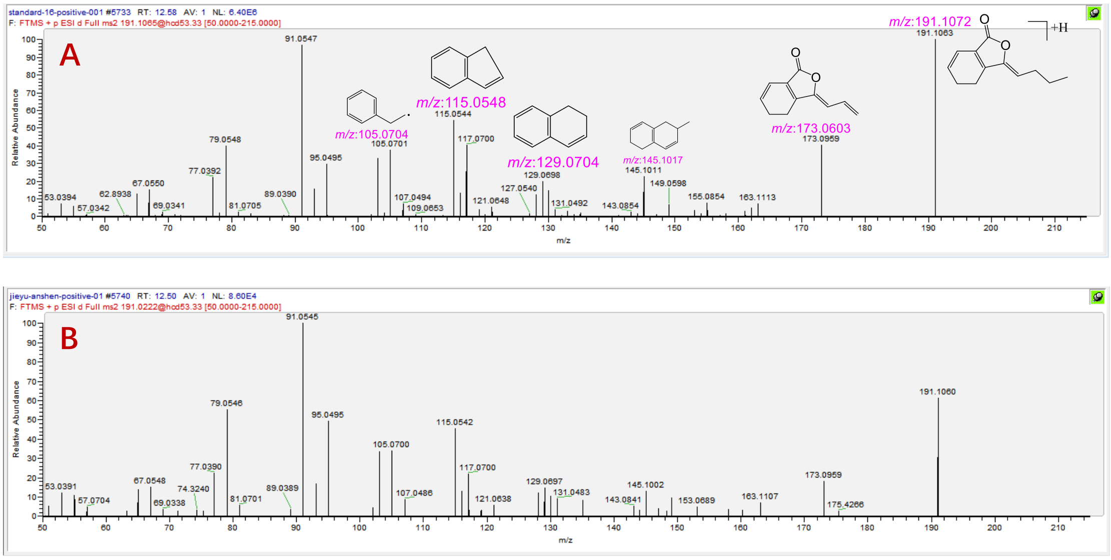

Fig. S35.1 The main results of standard ligustilide (CAS 81944-09-4, $C_{12}H_{14}O_2$) and its corresponding peak in the TIC diagram using UPLC-Q-Orbitrap-MS analysis. (**A**) The MS/MS fragments of standard ligustilide. (**B**) The MS/MS spectra from chromatographic peak in R.T. 12.50 min in the *Jieyu Anshen* Granule extract.

Note: The *m/z* values in purple are the calculated ones. The *m/z* calculation was based on the relative atomic masses of C (12.0000), H (1.007825), O (15.994915), and N (14.003074)[1].

Identification: As seen in Fig. S35.1, the R.T. value, molecular ion peak, MS/MS spectra, and characteristic pears were highly similar. Thus, the chromatographic peak in R.T. 12.50 min in the *Jieyu Anshen* Granule extract was identified as ligustilide (CAS 81944-09-4, $C_{12}H_{14}O_2$).

References:

[1] Gross., J.H., Mass spectrometry Beijing: Science press, 2013. 1.

Suppl. 36 Identification of jervine (CAS469-59-0, $C_{27}H_{39}NO_3$, M.W., 425.603).

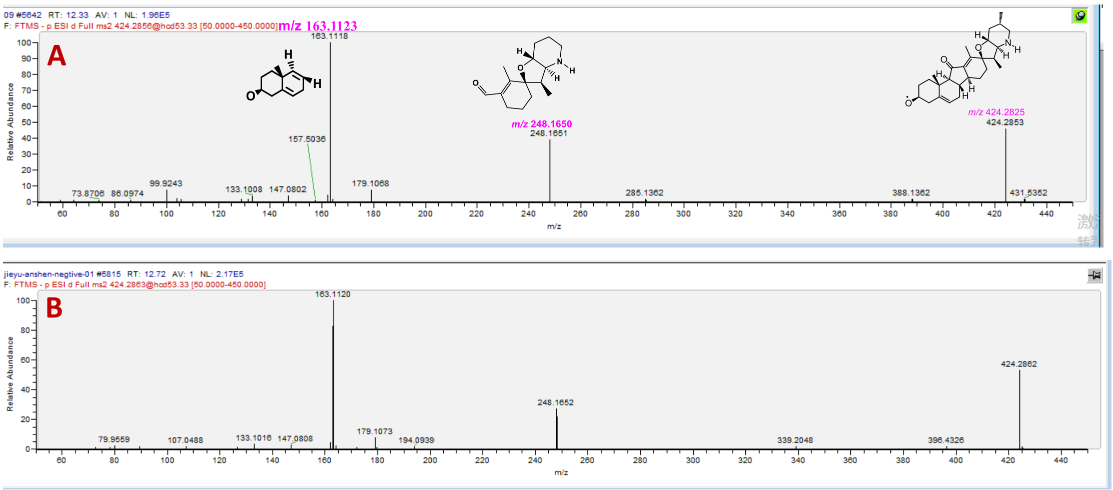

Fig. S36.1 The main results of standard jervine (CAS469-59-0, $C_{27}H_{39}NO_3$) and its corresponding peak in the TIC diagram using UPLC-Q-Orbitrap-MS analysis. (**A**) The MS/MS fragments of standard jervine. (**B**) The MS/MS spectra from chromatographic peak in R.T. 12.72 min in the *Jieyu Anshen* Granule extract.

Note: The *m/z* values in purple are the calculated ones. The *m/z* calculation was based on the relative atomic masses of C (12.0000), H (1.007825), O (15.994915), and N (14.003074)[1].

Identification: As seen in Fig. S36.1, the R.T. value, molecular ion peak, MS/MS spectra, and characteristic pears were highly similar. Thus, the chromatographic peak in R.T. 12.72 min in the *Jieyu Anshen* Granule extract was identified as jervine (CAS469-59-0, $C_{27}H_{39}NO_3$).

eferences:

[1] Gross., J.H., Mass spectrometry Beijing: Science press, 2013. 1.

Suppl. 37 Identification of tangeretin (CAS481-53-8, $C_{20}H_{20}O_7$, M.W.,372.369).

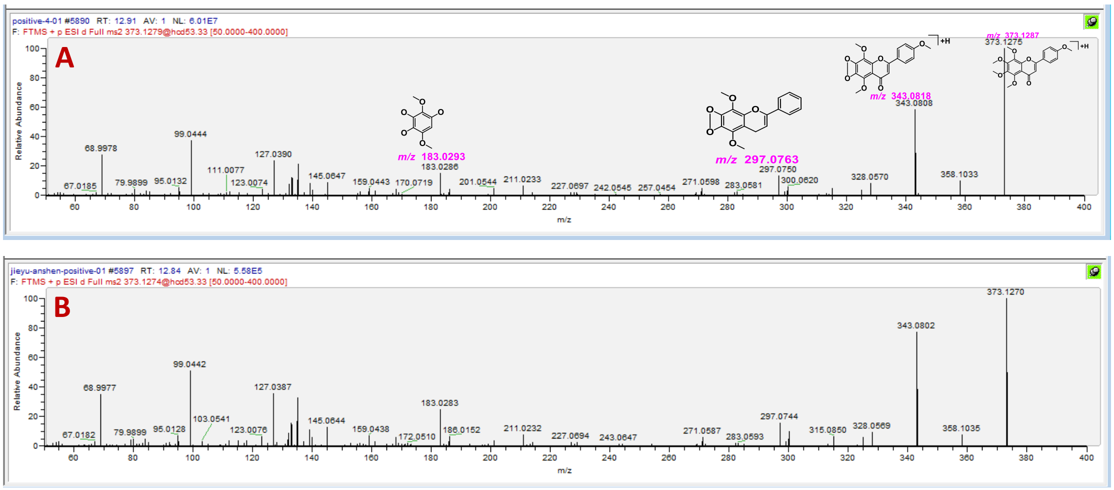

Fig. S37.1 The main results of standard tangeretin (CAS481-53-8, $C_{20}H_{20}O_7$) and its corresponding peak in the TIC diagram using UPLC-Q-Orbitrap-MS analysis. (**A**) The MS/MS fragments of standard tangeretin. (**B**) The MS/MS spectra from chromatographic peak in R.T. 12.84 min in the *Jieyu Anshen* Granule extract.

Note: The *m/z* values in purple are the calculated ones. The *m/z* calculation was based on the relative atomic masses of C (12.0000), H (1.007825), O (15.994915), and N (14.003074)[1].

Identification: As seen in Fig. S37.1, the R.T. value, molecular ion peak, MS/MS spectra, and characteristic pears were highly similar. Thus, the chromatographic peak in R.T. 12.84 min in the *Jieyu Anshen* Granule extract was identified as tangeretin (CAS481-53-8, $C_{20}H_{20}O_7$).

References:

[1] Gross., J.H., Mass spectrometry Beijing: Science press, 2013. 1.

Suppl. 38 Identification of glycyrrhizic acid (CAS 1405-86-3, $C_{42}H_{62}O_{16}$, M.W., 822.932).

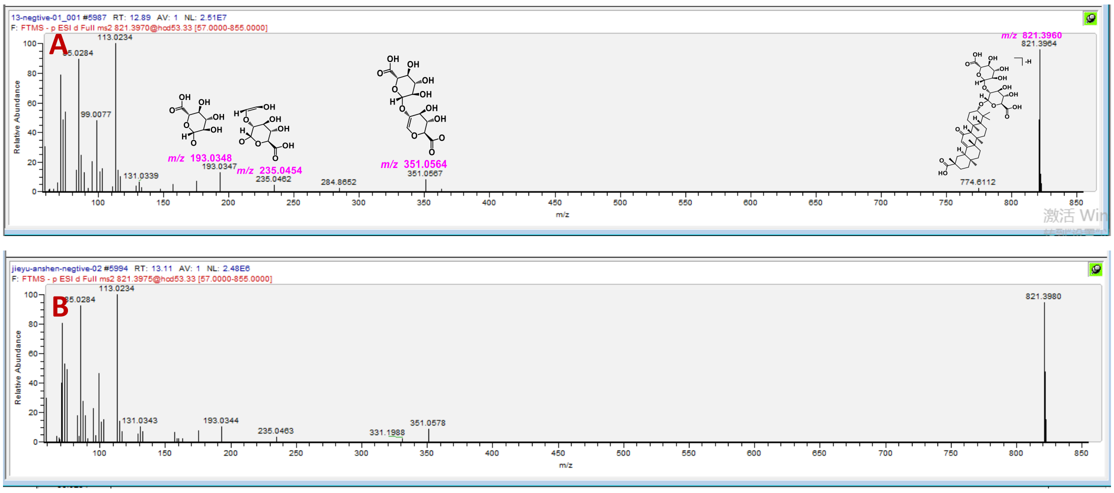

Fig. S38.1 The main results of standard glycyrrhizic acid (CAS 1405-86-3, $C_{42}H_{62}O_{16}$) and its corresponding peak in the TIC diagram using UPLC-Q-Orbitrap-MS analysis. (**A**) The MS/MS fragments of standard glycyrrhizic acid. (**B**) The MS/MS spectra from chromatographic peak in R.T. 13.11 min in the *Jieyu Anshen* Granule extract.

Note：The *m/z* values in purple are the calculated ones. The *m/z* calculation was based on the relative atomic masses of C (12.0000), H (1.007825), O (15.994915), and N (14.003074)[1].

Identification: As seen in Fig. S38.1, the R.T. value, molecular ion peak, MS/MS spectra, and characteristic pears were highly similar. Thus, the chromatographic peak in R.T. 13.11 min in the *Jieyu Anshen* Granule extract was identified as glycyrrhizic acid (CAS 1405-86-3, $C_{42}H_{62}O_{16}$).

References:

[1] Gross., J.H., Mass spectrometry Beijing: Science press, 2013. 1.

Suppl. 39 Identification of alantolactone (CAS 546-43-0, $C_{15}H_{20}O_2$)

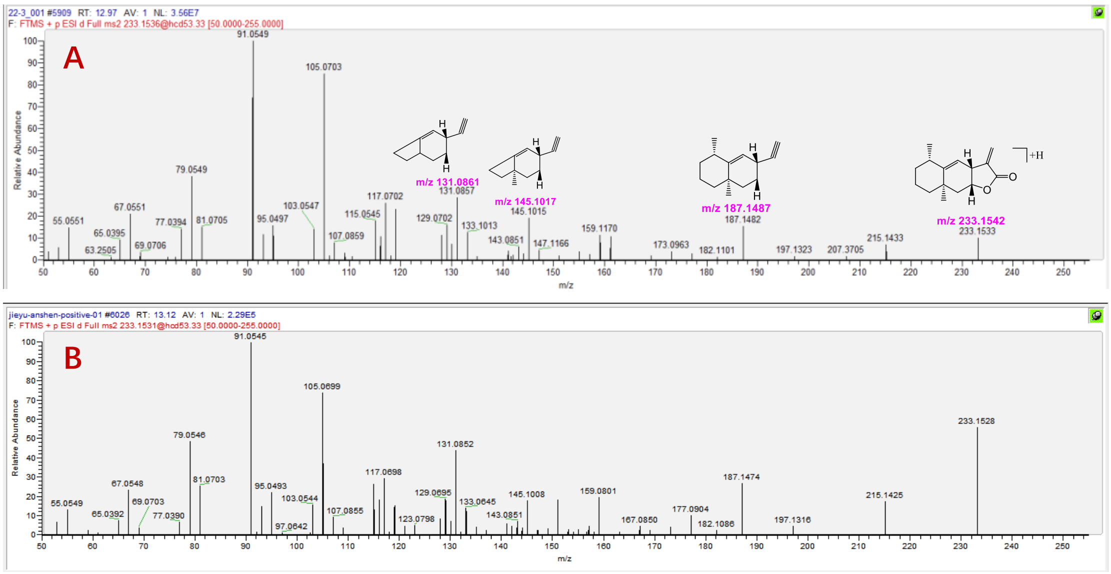

Fig. S39.1 The main results of standard alantolactone (CAS 546-43-0, $C_{15}H_{20}O_2$) and its corresponding peak in the TIC diagram using UPLC-Q-Orbitrap-MS analysis. (**A**) The MS/MS fragments of standard Alantolactone. (**B**) The MS/MS spectra from chromatographic peak in R.T. 13.12 min in the *Jieyu Anshen* Granule extract.

Note: The *m/z* values in purple are the calculated ones. The *m/z* calculation was based on the relative atomic masses of C (12.0000), H (1.007825), O (15.994915), and N (14.003074)[1].

Identification: As seen in Fig. S39.1, the R.T. value, molecular ion peak, MS/MS spectra, and characteristic pears were highly similar. Thus, the chromatographic peak in R.T. 13.12 min in the *Jieyu Anshen* Granule extract was identified as alantolactone (CAS 546-43-0, $C_{15}H_{20}O_2$).

References:

[1] Gross., J.H., Mass spectrometry Beijing: Science press, 2013. 1.

Suppl. 40 Identification of cyclocommunol (CAS 145643-96-5, $C_{20}H_{16}O_6$, M.W.,352.337).

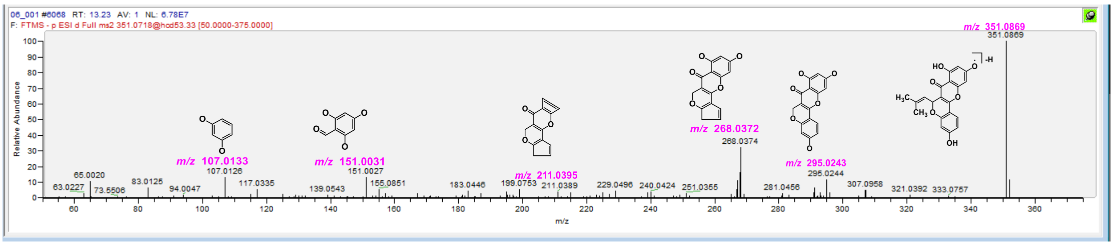

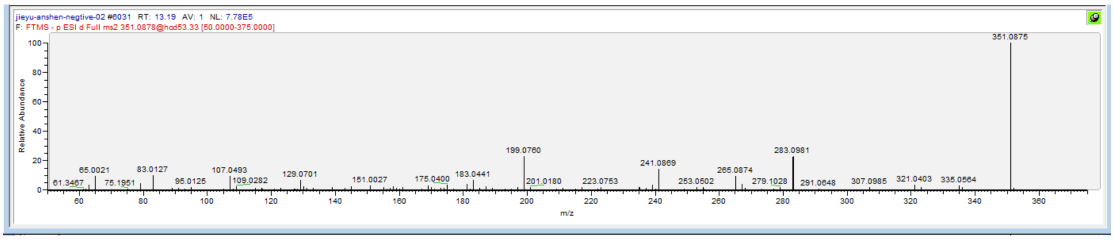

Fig. S40.1 The main results of standard cyclocommunol (CAS 145643-96-5, $C_{20}H_{16}O_6$) and its corresponding peak in the TIC diagram using UPLC-Q-Orbitrap-MS analysis. (**A**) The MS/MS fragments of standard cyclocommunol. (**B**) The MS/MS spectra from chromatographic peak in R.T. 13.19 min in the *Jieyu Anshen* Granule extract.

Note: The *m/z* values in purple are the calculated ones. The *m/z* calculation was based on the relative atomic masses of C (12.0000), H (1.007825), O (15.994915), and N (14.003074)[1].

Identification: As seen in Fig. S40.1, the R.T. value, molecular ion peak, MS/MS spectra, and characteristic pears were highly similar. Thus, the chromatographic peak in R.T. 13.19 min in the *Jieyu Anshen* Granule extract was identified as cyclocommunol (CAS 145643-96-5, $C_{20}H_{16}O_6$).

References:

[1] Gross., J.H., Mass spectrometry Beijing: Science press, 2013. 1.

Suppl. 41 Identification of 5-hydroxyflavone (CAS 491-78-1, $C_{15}H_{10}O_3$, M.W.238.238).

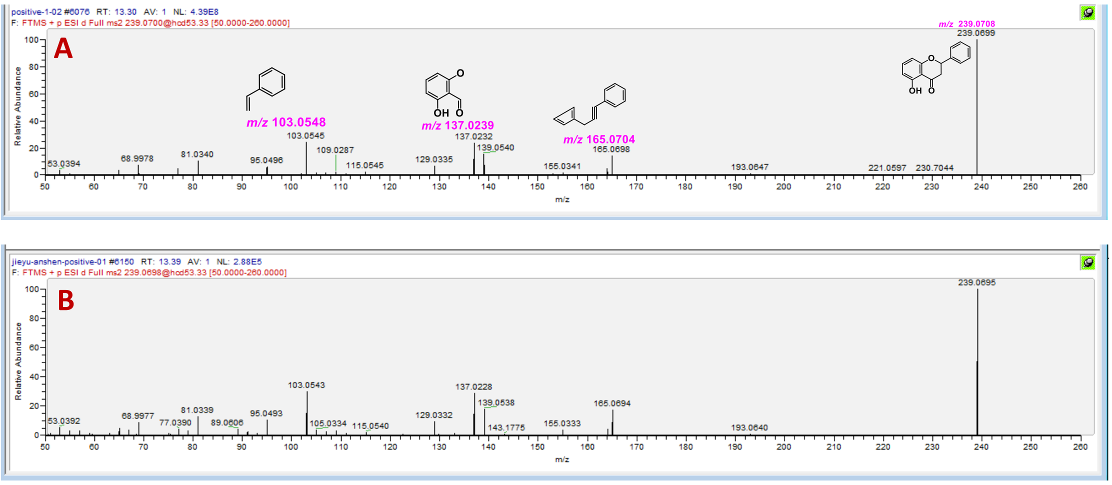

Fig. S41.1 The main results of standard 5-hydroxyflavone (CAS 491-78-1, $C_{15}H_{10}O_3$) and its corresponding peak in the TIC diagram using UPLC-Q-Orbitrap-MS analysis. (**A**) The MS/MS fragments of standard 5-hydroxyflavone. (**B**) The MS/MS spectra from chromatographic peak in R.T. 13.39 min in the *Jieyu Anshen* Granule extract.

Note: The *m/z* values in purple are the calculated ones. The *m/z* calculation was based on the relative atomic masses of C (12.0000), H (1.007825), O (15.994915), and N (14.003074)[1].

Identification: As seen in Fig. S41.1, the R.T. value, molecular ion peak, MS/MS spectra, and characteristic pears were highly similar. Thus, the chromatographic peak in R.T. 13.39 min in the *Jieyu Anshen* Granule extract was identified as 5-hydroxyflavone (CAS 491-78-1, $C_{15}H_{10}O_3$).

References:

[1] Gross., J.H., Mass spectrometry Beijing: Science press, 2013. 1.

Suppl. 42 Identification of saikosaponin A (CAS 20736-09-8, $C_{42}H_{68}O_{13}$, M.W., 780.982)

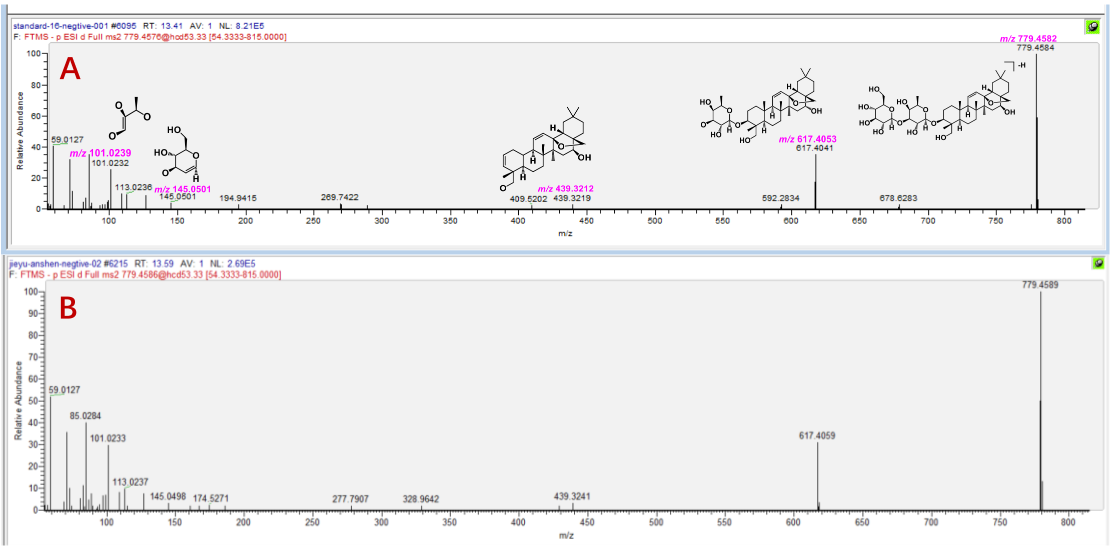

Fig. S42.1 The main results of standard saikosaponin A (CAS 20736-09-8, $C_{42}H_{68}O_{13}$) and its corresponding peak in the TIC diagram using UPLC-Q-Orbitrap-MS analysis. (**A**) The typical MS/MS fragments of standard saikosaponin A. (**B**) The typical MS/MS fragments of chromatographic peak in R.T. 13.59 min in the *Jieyu Anshen* Granule extract.

Note: The *m/z* values in purple are the calculated ones. The *m/z* calculation was based on the relative atomic masses of C (12.0000), H (1.007825), O (15.994915), and N (14.003074)[1].

Identification: As seen in Fig. S42.1, the R.T. value, molecular ion peak, MS/MS spectra, and characteristic pears were highly similar. Thus, the chromatographic peak in R.T. 13.59 min in the *Jieyu Anshen* Granule extract was identified as saikosaponin A (CAS 20736-09-8, $C_{42}H_{68}O_{13}$).

References:

[1] Gross., J.H., Mass spectrometry Beijing: Science press, 2013. 1.

Suppl. 43 Identification of saikosaponin D (CAS20874-52-6, $C_{42}H_{68}O_{13}$, M.W. 780.982）.

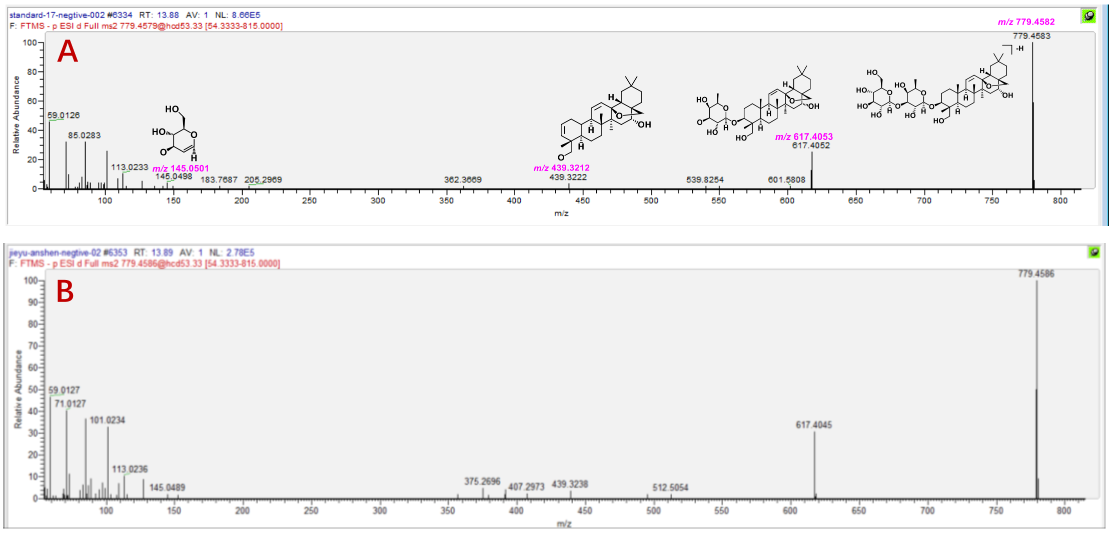

Fig. S43.1 The main results of standard saikosaponin D （CAS 20874-52-6, $C_{42}H_{68}O_{13}$） and its corresponding peak in the TIC diagram using UPLC-Q-Orbitrap-MS analysis. (**A**) The MS/MS fragments of standard saikosaponin D. (**B**) The MS/MS spectra from chromatographic peak in R.T. 13.89 min in the *Jieyu Anshen* Granule extract.

Note: The *m/z* values in purple are the calculated ones. The *m/z* calculation was based on the relative atomic masses of C (12.0000), H (1.007825), O (15.994915), and N (14.003074)[1].

Identification: As seen in Fig. S43.1, the R.T. value, molecular ion peak, MS/MS spectra, and characteristic pears were highly similar. Thus, the chromatographic peak in R.T. 13.89 min in the *Jieyu Anshen* Granule extract was identified as saikosaponin D （CAS 20874-52-6, $C_{42}H_{68}O_{13}$）

References:

[1] Gross., J.H., Mass spectrometry Beijing: Science press, 2013. 1.

Suppl. 44 Identification of 18β-glycyrrhetinic acid (CAS 471-53-4, $C_{30}H_{46}O_4$)

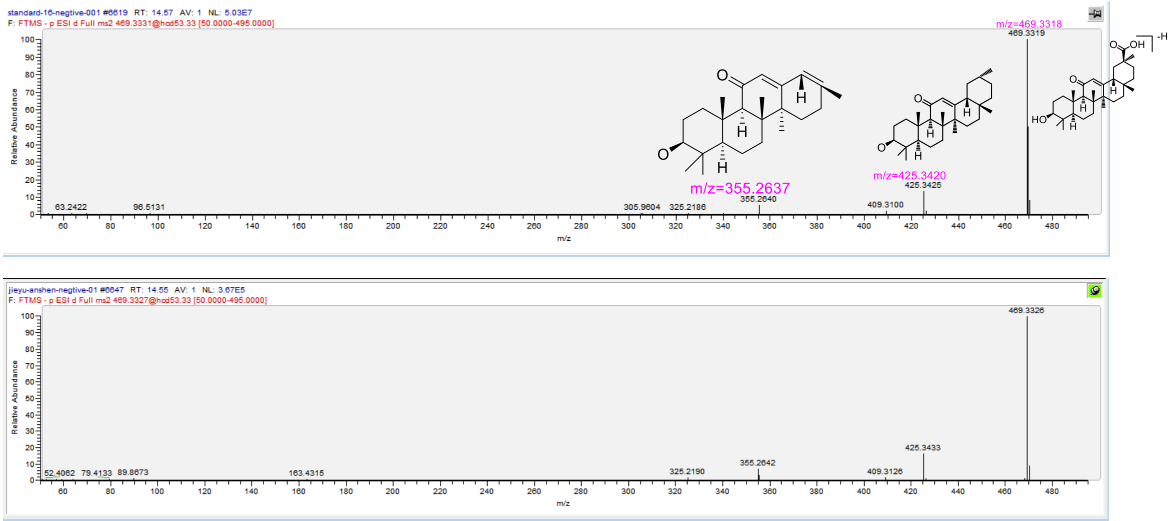

Fig. S44.1 The main results of standard 18β-glycyrrhetinic acid (CAS 471-53-4, $C_{30}H_{46}O_4$) and its corresponding peak in the TIC diagram using UPLC-Q-Orbitrap-MS analysis. (**A**) The MS/MS fragments of standard 18β-glycyrrhetinic acid. (**B**) The MS/MS spectra from chromatographic peak in R.T. 14.55 min in the *Jieyu Anshen* Granule extract.

Note: The *m/z* values in purple are the calculated ones. The *m/z* calculation was based on the relative atomic masses of C (12.0000), H (1.007825), O (15.994915), and N (14.003074)[1].

Identification: As seen in Fig. S44.1, the R.T. value, molecular ion peak, MS/MS spectra, and characteristic pears were highly similar. Thus, the chromatographic peak in R.T. 14.55 min in the *Jieyu Anshen* Granule extract was identified as 18β-glycyrrhetinic acid (CAS 471-53-4, $C_{30}H_{46}O_4$).

References:

[1] Gross., J.H., Mass spectrometry Beijing: Science press, 2013. 1.

Suppl. 45 Identification of ethyl stearate (CAS111-61-5, $C_{20}H_{40}O_2$, M.W., 312.53).

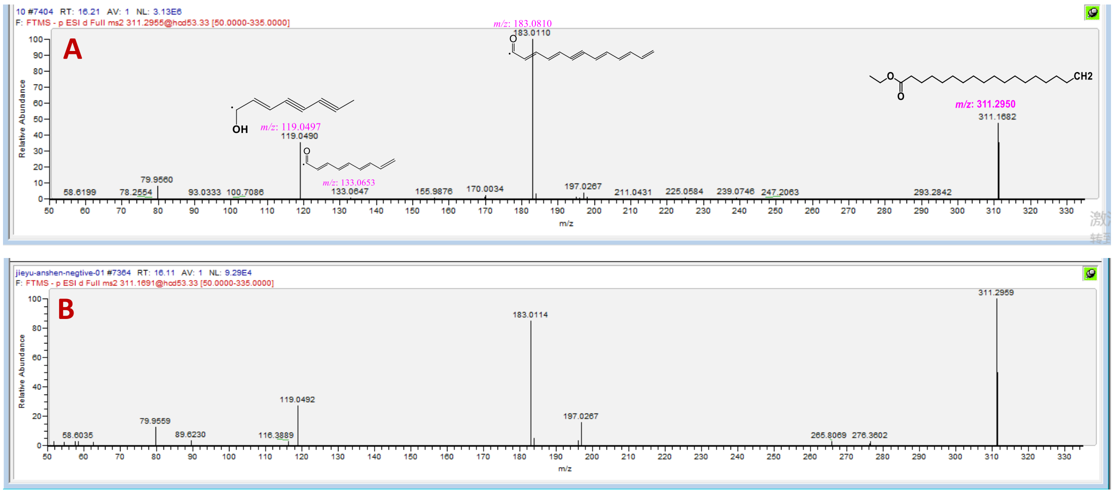

Fig. S45.1 The main results of standard ethyl stearate (CAS111-61-5, $C_{20}H_{40}O_2$) and its corresponding peak in the TIC diagram using UPLC-Q-Orbitrap-MS analysis. (**A**) The MS/MS fragments of standard ethyl stearate. (**B**) The MS/MS spectra from chromatographic peak in R.T. 16.11 min in the *Jieyu Anshen* Granule extract.

Note: The *m/z* values in purple are the calculated ones. The *m/z* calculation was based on the relative atomic masses of C (12.0000), H (1.007825), O (15.994915), and N (14.003074)[1].

Identification: As seen in Fig. S45.1, the R.T. value, molecular ion peak, MS/MS spectra, and characteristic pears were highly similar. Thus, the chromatographic peak in R.T. 16.11 min in the *Jieyu Anshen* Granule extract was identified as ethyl stearate (CAS111-61-5, $C_{20}H_{40}O_2$).

References:

[1] Gross., J.H., Mass spectrometry Beijing: Science press, 2013. 1.

Suppl. 46 Identification of hypericin (CAS 548-04-9, $C_{30}H_{16}O_8$)

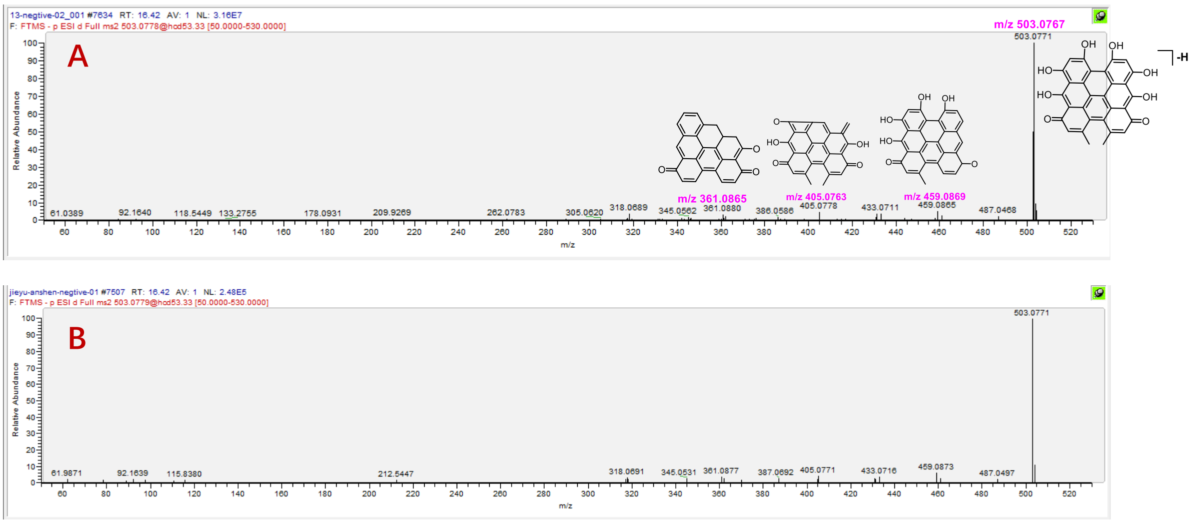

Fig. S46.1 The main results of standard hypericin (CAS 548-04-9, $C_{30}H_{16}O_8$) and its corresponding peak in the TIC diagram using UPLC-Q-Orbitrap-MS analysis. (**A**) The MS/MS fragments of standard hypericin. (**B**) The MS/MS spectra from chromatographic peak in R.T. 16.42 min in the *Jieyu Anshen* Granule extract.

Note: The *m/z* values in purple are the calculated ones. The *m/z* calculation was based on the relative atomic masses of C (12.0000), H (1.007825), O (15.994915), and N (14.003074)[1].

Identification: As seen in Fig. S46.1, the R.T. value, molecular ion peak, MS/MS spectra, and characteristic pears were highly similar. Thus, the chromatographic peak in R.T. 16.42 min in the *Jieyu Anshen* Granule extract was identified as hypericin (CAS 548-04-9, $C_{30}H_{16}O_8$).

References:

[1] Gross., J.H., Mass spectrometry Beijing: Science press, 2013. 1.

Suppl. 47 Identification of (+)-4-cholesten-3-one (CAS601-57-0, $C_{27}H_{44}O$, M.W., 384.638).

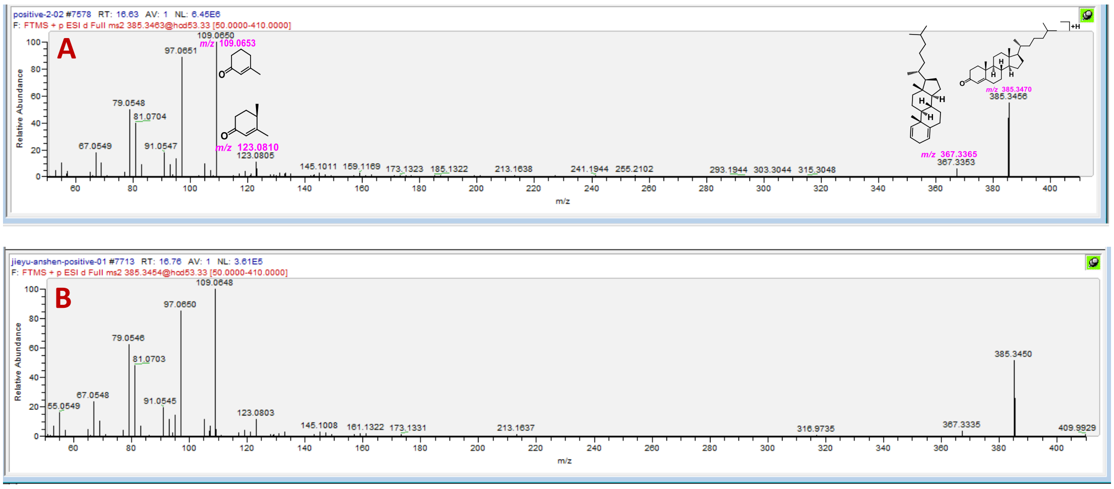

Fig. S47.1 The main results of standard (+)-4-cholesten-3-one (CAS601-57-0, $C_{27}H_{44}O$) and its corresponding peak in the TIC diagram using UPLC-Q-Orbitrap-MS analysis. (**A**) The MS/MS fragments of standard (+)-4-cholesten-3-one. (**B**) The MS/MS spectra from chromatographic peak in R.T. 16.76 min in the *Jieyu Anshen* Granule extract.

Note: The *m/z* values in purple are the calculated ones. The *m/z* calculation was based on the relative atomic masses of C (12.0000), H (1.007825), O (15.994915), and N (14.003074)[1].

Identification: As seen in Fig. S47.1, the R.T. value, molecular ion peak, MS/MS spectra, and characteristic pears were highly similar. Thus, the chromatographic peak in R.T. 16.76 min in the *Jieyu Anshen* Granule extract was identified as (+)-4-cholesten-3-one (CAS 601-57-0, $C_{27}H_{44}O$).

References:

[1] Gross., J.H., Mass spectrometry Beijing: Science press, 2013. 1.
